# An Online experiment during the 2020 US–Iran crisis shows that exposure to common enemies can increase political polarization

**DOI:** 10.1038/s41598-022-23673-0

**Published:** 2022-11-11

**Authors:** Eaman Jahani, Natalie Gallagher, Friedolin Merhout, Nicolo Cavalli, Douglas Guilbeault, Yan Leng, Christopher A. Bail

**Affiliations:** 1 Department of Statistics, University of California, Berkeley, 367 Evans Hall, Berkeley, CA 94720-3860 USA; 2 Department of Psychology, Princeton University, South Dr, Princeton, NJ 08540 USA; 3 Department of Sociology, University of Copenhagen, 1353 Copenhagen K, Denmark; 4 Center for Social Data Science, University of Copenhagen, Øster Farimagsgade 5, 1353 Copenhagen, Denmark; 5 Carlo F. Dondena Centre, Bocconi University, 1 Via Guglielmo Röntgen, 20136 Milan, Italy; 6 Nuffield College and Department of Sociology, Oxford University, 1 New Road, Oxford, OX1 1NF UK; 7 Haas School of Business, University of California, Berkeley, 2220 Piedmont Ave, Berkeley, CA 94720 USA; 8 McCombs School of Business, University of Texas at Austin, 300 MLK Jr., Austin, TX 78712 USA; 9 Department of Sociology, Duke University, 254 Soc. Psych Hall, Durham, NC 27708 USA; 10 Sanford School of Public Policy, Duke University, Durham, NC 27708 USA

**Keywords:** Human behaviour, Information technology

## Abstract

A longstanding theory indicates that the threat of a common enemy can mitigate conflict between members of rival groups. We tested this hypothesis in a pre-registered experiment where 1670 Republicans and Democrats in the United States were asked to complete an online social learning task with a bot that was labeled as a member of the opposing party. Prior to this task, we exposed respondents to primes about (a) a common enemy (involving Iran and Russia); (b) a patriotic event; or (c) a neutral, apolitical prime. Though we observed no significant differences in the behavior of Democrats as a result of priming, we found that Republicans—and particularly those with very strong conservative views—were significantly *less* likely to learn from Democrats when primed about a common enemy. Because our study was in the field during the 2020 Iran Crisis, we were able to further evaluate this finding via a natural experiment—Republicans who participated in our study after the crisis were even less influenced by the beliefs of Democrats than those Republicans who participated before this event. These findings indicate common enemies may not reduce inter-group conflict in highly polarized societies, and contribute to a growing number of studies that find evidence of asymmetric political polarization in the United States. We conclude by discussing the implications of these findings for research in social psychology, political conflict, and the rapidly expanding field of computational social science.

## Introduction

Political polarization—or the tendency for members of rival political groups to adopt increasingly distant opinions about how to solve social problems—is pervasive in many Western Democracies today^1–4^. In the United States, for example, 59.3% of Democratic voters believe federal aid to the poor should be increased compared to only 20.2% of Republicans voters. Conversely, 68.9% of Republicans believe immigration to the United States should be decreased, compared to 21.9% of Democrats^1^. Such discrepancies extend beyond policy issues into the attitudes of Republicans and Democrats towards each other. The proportion of Americans identified with a political party who would be uncomfortable if their child married someone of the opposite party has risen from less than 10% in 1960 to at least 33% in 2010^2^. These trends show no signs of slowing in the wake of the recent divisive presidential elections and the impeachment trials of former President Donald Trump.

Though social scientists have offered many explanations for the recent growth of political polarization, relatively little research has identified solutions to this increasingly urgent social problem^3^. One possibility is that members of rival groups will set aside their differences if they face a shared threat from a common enemy^4–6^. This theory—which has roots in social psychology, social network analysis, and philosophy that date back as far as a Sanskrit treatise on warfare from the fourth century B.C.—has been invoked to explain the consolidation of rival factions and even the emergence of the modern nation-state in Western Europe and many other places^7–11^. Indeed, this theory is so well-established that it has reached widespread prominence in lay beliefs of intergroup dynamics, as captured by the popular proverb: “the enemy of my enemy is my friend”^9^.

Different proposals have been offered to explain this phenomenon. Within the common ingroup identity model^10^, this effect occurs because members of rival factions come to realize they have more in common with each other than their shared enemy. In the United States, for example, some argue that the threat of the Soviet Union prevented political polarization throughout the Cold War because it fostered a sense of shared fate or national identity that inspired Americans to set aside their differences in the face of a formidable enemy^11–14^. Yet it is also possible that simply priming national identity might have a similar effect, if the mechanism of depolarization is reminding rival factions about their similarities to each other^7,10^. Another possibility is that the common enemy effect is driven by fear of out-groups, or some combination of this process and ingroup favoritism^11–14^.

However, a series of recent studies have provided empirical evidence that runs contrary to the theory that common enemies—by activating shared superordinate identities—bring rival groups together. Dach-Gruschow and Hong^15^, for instance, find that identifying with the common superordinate identity of “American” failed to unite white and Black Americans in the United States in the aftermath of Hurricane Katrina. Klar^16^ builds on this by showing experimentally that priming Republican and Democratic women to identify with each other as “women” (the superordinate gender category) actually led to the amplification of cross-party biases. Klar explains these counterintuitive results using a theory put forward by Rutchik and Eccleston^17^, who argue that “when there is a perception that subgroups do not have a shared conception of the superordinate group, appeals to the common ingroup identity made by outgroup members are likely to backfire” (111). This theory identifies a plausible mechanism for why exposure to common enemies may fail to unite rival groups.

Importantly, there are several reasons to suspect that the backfire effects of common enemy priming are especially likely to hold in the current political landscape of the U.S., which is characterized by high cross-party animosity. We expect that priming superordinate identities via common enemy priming can backfire when animosity among rival political groups is high, since these are conditions in which rival groups are especially likely to disagree on how they conceptualize their shared superordinate identity, as well as who is considered as belonging to it. In particular, we maintain that when rival groups are sufficiently antagonistic, they may dislike and distrust each other as much if not more than the common enemy, such that the common ingroup identity linking rival groups may feel like a threat to the existing partisan identity; under such conditions, priming a common enemy threat may inadvertently decrease social learning and cooperation across rival groups^7,16,17^. This framework is especially well-suited for characterizing the current tensions between the Republican and Democratic parties in the U.S., given recent survey research^18^ indicating that both parties consistently view each other as “un-American” and as a “threat to the nation”. For this reason, exposure to a common enemy that threatens Democrats and Republicans equally as “Americans” may backfire by decreasing cross-party social learning and cooperation.

This reasoning also highlights why exposure to common enemies may be a particularly potent way of exacerbating partisan tensions, beyond priming superordinate identities in other more neutral or even positive ways. Exposure to common enemies can unearth aggravating differences among rival groups in terms of each group’s relation to the common enemy, thereby revealing a vital dimension of difference in how each group perceives their superordinate identity and who belongs to it. In other words, exposure to common enemies can intensify partisan differences and exclusion in the definition of superordinate identities by, for example, priming the belief that one’s rival group is allied with the common enemy, with the effect of disqualifying them from the superordinate identity and framing them as yet another enemy. For this reason, common enemy priming may be especially effective at priming divisive perceptions of shared superordinate identities, whereas more positive ways of priming superordinate identities (such as priming the identity of “American” by evoking Fourth of July celebrations) may be biased toward activating construals of shared identity that skew toward inclusiveness—a difference that may play a significant role in why prior efforts to prime superordinate identities, which focus primarily on positive priming mechanisms, have shown resulting reductions in cross-party bias and animosity^18^.

Furthermore, there are two key reasons to expect that the backfire effects of common enemies may not be uniformly distributed across rival groups and that they may be especially strong among Republicans in the U.S. context. First, a fairly recent nationally representative survey indicates that Republicans are significantly more likely to identify Democrats as “un-American” and as a “threat to the nation” than the reverse^19^; specifically, 27% of Democrats viewed Republicans as a threat to the nation’s well-being, whereas 36% of Republicans viewed Democrats as a threat to the nation’s well-being, marking a sizable 9 percentage point difference^19^. Second, these survey results are consistent with a broader body of work demonstrating that Republicans react more strongly to threats concerning partisan and national identities, which prior studies account for through a variety of psychological mechanisms, including Republicans’ greater propensity toward patriotism^20,21^. Relatedly, a number of studies document asymmetric polarization, whereby inter-group animosity appears to be driven more by Republicans than Democrats across a range of contexts^22–25^. If, as Rutchik and Eccleston^17^ and Klar^16^ propose, attempts to prime unifying identities can backfire when rival groups differ substantially in their views of their shared identities, then it follows that these backfire effects may be particularly strong among Republicans, who are especially prone to characterizing Democrats as thinking differently about American identity and as ultimately not belonging to it.

Studying whether common enemies reduce political polarization presents numerous methodological challenges. To begin, external threats are not randomly distributed across countries or historical contexts and rigorous causal inference is thus not possible with observational data. Similarly, field experiments that expose members of rival groups to common enemies would either be unethical, logistically impossible, or both. Simple survey experiments, however, lack the external validity necessary to demonstrate whether exposure to a common enemy shapes anything more than ephemeral attitudes or dispositions. In this paper, we adopt a hybrid research design in which we recruited a large group of Democrats and Republicans to participate in a social learning task^24,26^ which we developed using the online platform Empirica^27^ to study how different primes about collective identity influence how partisans exchange information to collaboratively solve an estimation problem when financial incentives are at stake.

## Methods

**Figure 1:**
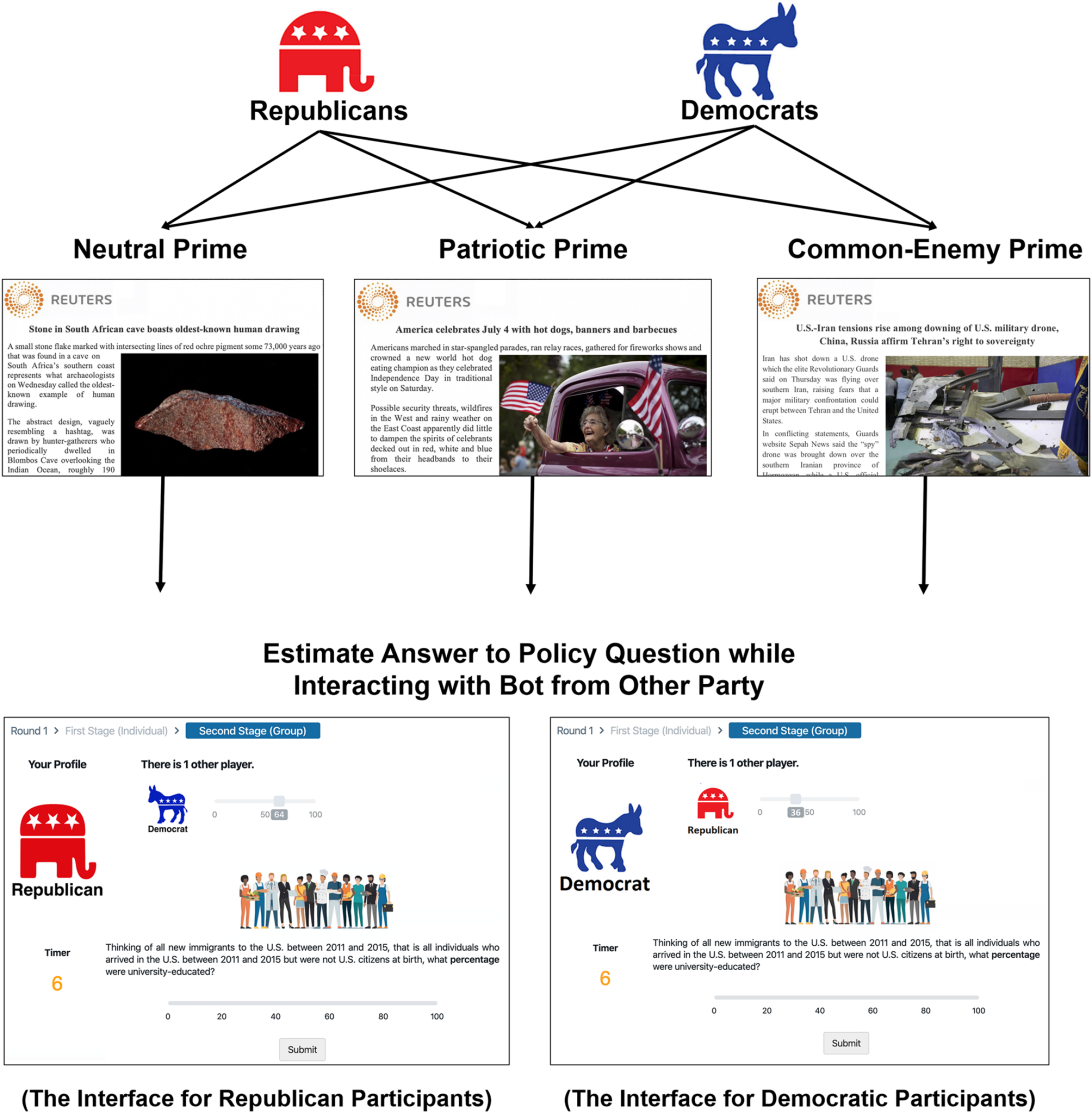
Schematic illustrating the experimental design. 1670 Republicans and Democrats were randomized into one of three experimental conditions: (1) the neutral prime condition, where they read an article about early human carvings in South Africa; (2) a patriotic prime condition, where they read an article about Fourth of July celebrations; and (3) a common-enemy prime condition, where they read an article about the combined threat of Iran, China, and Russia. After reading the article, each participant was offered financial incentives to estimate the answer to a question about a political issue and told that their compensation would increase according to the accuracy of their response. After submitting their first estimate, participants were shown the estimate of a bot impersonating a member of the opposing party. By measuring how often members of each party revise their answers towards the bot in the subsequent round of estimation, we measure how much members of each party learn from the opposing party within each treatment condition.

This research was approved by the Institutional Review Board at Northwestern University, where the study was conducted, and it included informed consent by all participants. All methods were carried out in accordance with the relevant guidelines and regulations specified by Northwestern’s Institutional Review Board. Figure 1 (below) describes our research design. From October 2019 to January 2020, we recruited 1670 self-identified Republicans or Democrats who live in the United States from an online panel to complete a brief survey about their political preferences. Participants were randomized into one of three conditions. In the first condition, respondents were asked to read a neutral or apolitical article about early human drawings that were recently discovered by archaeologists in South Africa. This condition serves as our control population. In the second condition, we study the effect of priming ingroup identity alone by asking respondents to read an article about Fourth of July celebrations in several U.S. cities; the ingroup identity being primed by this article is the shared superordinate identity of “American”. In the third condition, we exposed respondents to a common enemy prime in which they read an article about how Russia, Iran, and China were conspiring to attack U.S. military and political interests. As our Supplementary Materials describe, these articles were carefully selected from a group of 43 candidate primes from *Reuters.com* that we pretested in order to ensure they created the expected priming effect among both Republicans and Democrats. We elected to use articles from *Reuters.com* because previous studies indicate it is equally trusted and well-respected by Republicans and Democrats^25^.

To collect a behavioral measure of political polarization, we told each respondent they could receive additional pay for providing more accurate estimates to a question about a political issue: “What percentage of immigrants between 2011 and 2015 were college educated?” Respondents first estimated the answer to this question themselves, with no further input. After making their own estimate, they were exposed to the guess of a bot which impersonated a member of the opposing political party who was also involved in the estimation task. The bot always provided an initial estimate that was about 50 percentage points away from the participant’s initial guess. After viewing this response, participants were invited to revise their estimate. Below, we report how much participants revised their predictions after being exposed to an estimate that they believed was from a member of the opposing political party. The extent to which each respondent updated their estimate towards that of the bot describes the degree to which participants learned from a member of the opposing party and were willing to cooperatively incorporate their views into their own, when financial incentives are at stake.

To further validate our proposed mechanism—namely that the common enemy article primed the salience of the polarized superordinate identity of “American”—we conducted an exit survey across all conditions that participants completed immediately after the experiment, in which participants were asked to identify the extent to which they identify as being “American”, as well as the extent to which they identify with their own and the opposing party.

We gained additional information about the effects of common enemy priming via a natural experiment that occurred during our fieldwork^28^. On January 3rd, 2020, United States special forces in Iraq assassinated Qassim Suleimani, an influential Iranian general. This triggered a major geopolitical crisis that many people believed might have caused the outbreak of war between the two countries. This event occurred in the middle of our fieldwork, which began in October 2019 and concluded in late January 2020. Since our common enemy prime involved discussion of US–Iran relations, this unanticipated exogenous event gave us additional leverage to test how increasing the salience of a common enemy interacts with extant partisan tensions. If the saliency of a common enemy can exacerbate polarization—and particularly among Republicans—in highly polarized contexts, we should find that this exogenous shock—which increased the salience of Iran as a common enemy—should similarly exacerbate these backfire effects. Since numerous features of this natural exogenous shock were beyond our methodological control, we are limited in our ability to exploit this shock to highlight particular mechanisms driving changes in participants’ responses; such mechanisms may, for instance, include biases concerning the way in which this shock was covered in the media, along with selection biases in which audiences were most likely to encounter these media narratives. Identifying the key channels through which this shock influenced participants' responses is outside the scope of this study; for our purposes, the Iran Crisis served as a natural instrument for validating the robustness of our predictions concerning the backfire effects of common enemy priming. In other words, this theory was falsifiable with respect to this natural shock: if the Iran crisis had no effect or the opposite effect on the willingness for Democrats and Republicans to influence each other’s beliefs, then this would speak against the prediction that increasing the salience of common enemies in polarized contexts exacerbates polarized beliefs and behaviors.

## Results

**Figure 2:**
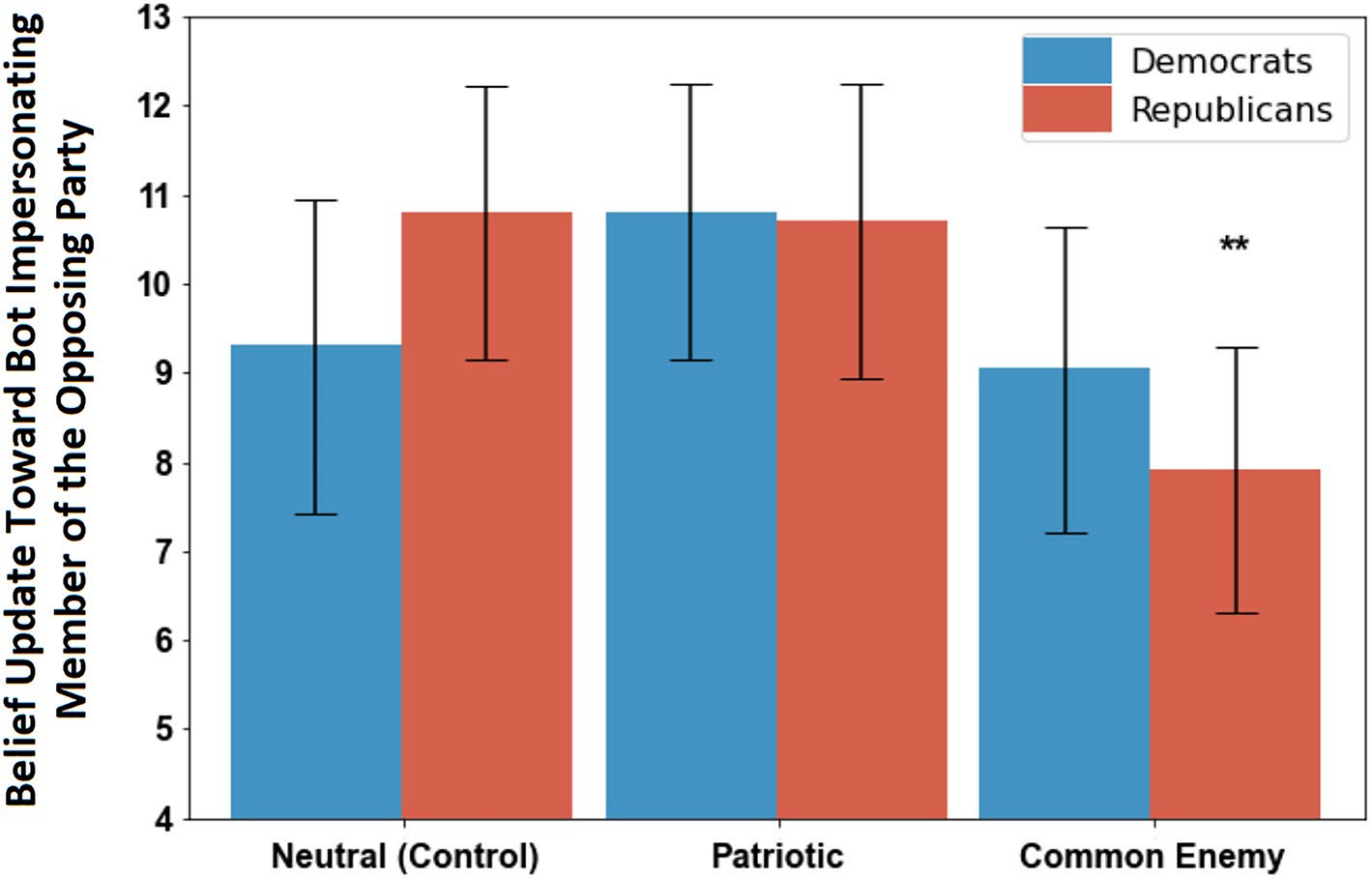
The extent that participants updated their beliefs toward the opinion of the bot impersonating a member of the opposing party during the collaborative online task, shown across experimental conditions and differentiated by political party. Vertical axis describes the post-stratified average belief update (in percentage points) for participants in each priming condition, where strata are defined by gender, political knowledge, the accuracy of initial guess, and awareness of bot’s membership in the opposing party. Larger values indicate that the participant updated their beliefs to become closer to the bot’s opinions, demonstrating greater receptivity to social influence from the other party. The neutral condition provides a baseline of comparison or a “control” condition. Error bars display 95% confidence intervals. **p < 0.01. See SI or description of our post-stratification methodology.

We estimate the effect of exposure to a common enemy and patriotic prime, respectively, by comparing whether people in these two conditions updated their beliefs more or less towards the bot impersonating the opposing party than those in the neutral, control condition. As Fig. 2 shows, we observed no significant differences in the willingness of Democrats to update their estimates towards the bot impersonating a Republican respondent in the study across each priming condition (*p* > 0.05, N = 530). In contrast, Republican participants were significantly less likely to be influenced by the bot—which they believed to be a Democrat—after exposure to the common enemy prime, as compared to Republicans in the control condition (*p* < 0.01, N = 314).

**Figure 3:**
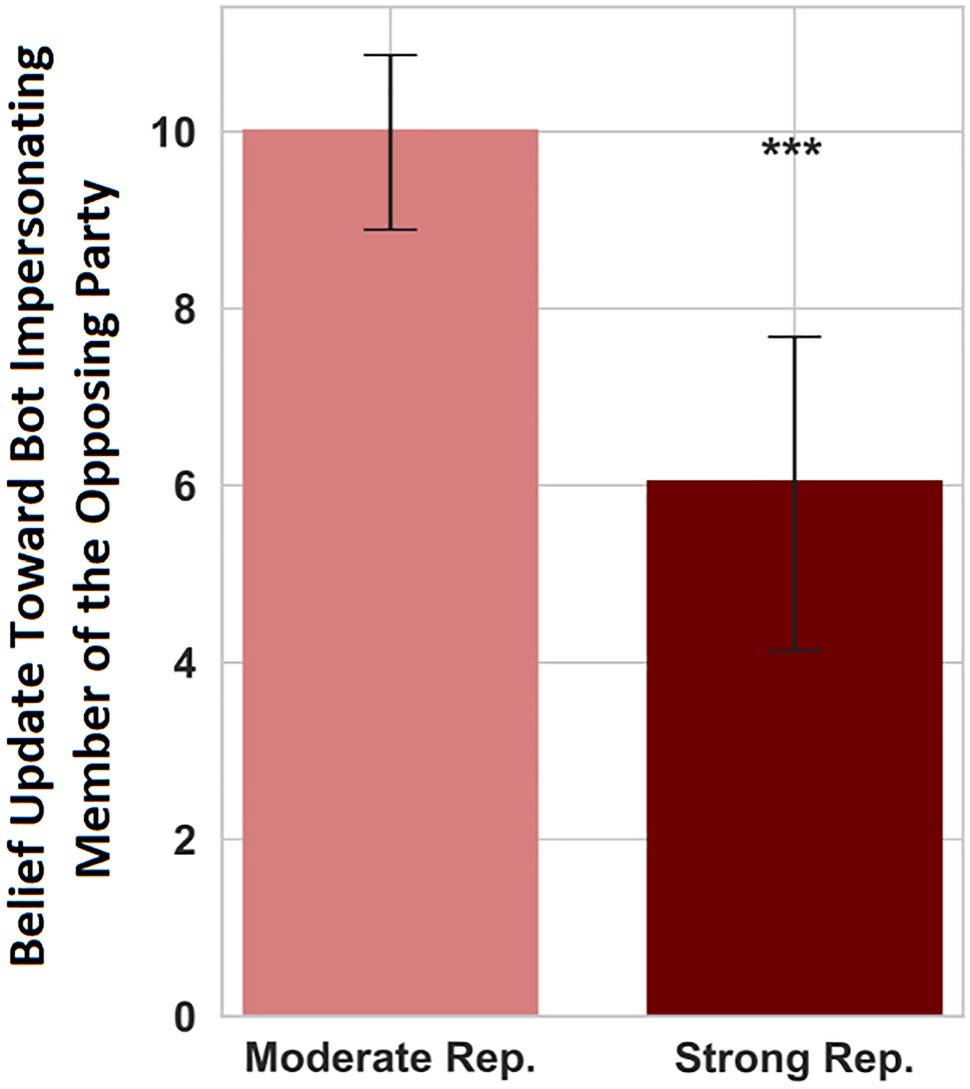
The extent that Republicans updated their beliefs toward the opinion of the Democrat bot during the online task across experimental conditions, split by the strength of partisanship. The data are collapsed across conditions. Vertical axis describes the post-stratified average belief update (in percentage points), where strata are defined by gender, political knowledge, the accuracy of initial guess, and awareness of bot’s membership in the opposing party. Larger values indicate that the participant updated their beliefs to become closer to the bot’s opinions, demonstrating greater receptivity to social influence from the other party. Strong Republicans (Rep.) are defined as those who are in the top 10th percentile of partisan identification measured by feeling thermometers. Moderate Republicans (Rep.) are those in all other percentiles. These “moderate republicans” provide a baseline of comparison. Error bars display 95% confidence intervals. ***p < 0.001. See SI for description of our post-stratification methodology.

What is more, the strength of partisanship among Republicans exacerbated this effect. As Fig. 3 shows, Republican respondents who were in the top 10th percentile of a thermometer-based measure of ingroup favoritism were less likely to update towards the bot impersonating a Democrat than those with less strong partisan views (*p* < 0.001, N = 484) (see SI for details on this thermometer measure). We observed no significant differences among Democrats using the same strength of partisanship measure. Supplementary analyses show that these results hold under a number of robustness tests and under alternative methods of measuring the strength of partisanship (Fig. S5).

Supplementary analyses support our proposed mechanism (SI). Our exit survey indicates that Republicans exposed to the common enemy prime identified significantly more strongly with being “American” than Republicans in the control condition (Fig. S6A). Yet this increase in identification with being “American” among Republicans did not include a significant change in Republicans’ willingness to identify with Democrats, suggesting an exclusive conception of American identity (Fig. S6B). By contrast, Democrats exposed to the common enemy article were less likely to identify as being “American” than Democrats in the control condition (Fig. S7). These results lend further support to our findings regarding asymmetric polarization, which suggest that Republicans and Democrats differed in their conception of American identity and its relation to the common enemy threat, and that an exclusive conception of American identity was activated among Republicans in particular, consistent with recent data from a nationally representative survey^18^ indicating that Republicans are especially likely to identify Democrats as un-American and as threats to the nation.

**Figure 4:**
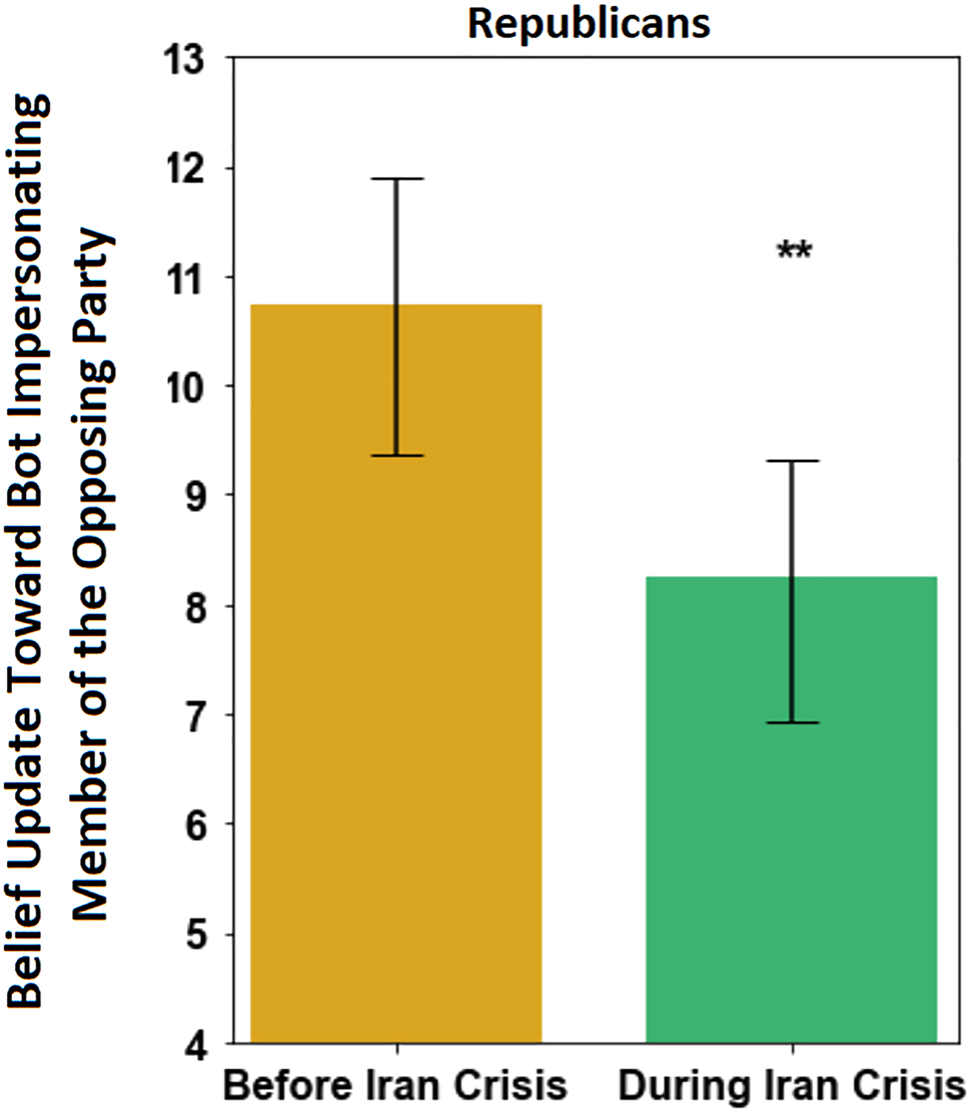
The extent that Republicans updated their beliefs toward the opinion of the Democrat bot during the collaborative online task across experimental conditions, before and after the 2020 Iran crisis. The data are collapsed across conditions. Vertical axis describes the post-stratified average belief update (in percentage points), where strata are defined by gender, political knowledge, the accuracy of initial guess, and awareness of bot’s membership in the opposing party. Larger values indicate that the participant updated their beliefs to become closer to the bot’s opinions, demonstrating greater receptivity to social influence from the other party. The “Before Iran Crisis” outcomes provide a baseline of comparison. Error bars display 95% confidence intervals. **p < 0.01. See SI for description of our post-stratification methodology.

Lastly, we examine the willingness of participants to use information provided by a member of the other party after a natural shock which occurred during our fieldwork and was expected to amplify the salience of our common enemy prime. In the middle of our fieldwork, on January 3rd, 2020, United States special forces in Iraq assassinated Qassim Suleimani, an influential Iranian general, triggering widespread panic about the possibility of war. Since our common enemy prime depicted Iran as a common enemy, this unanticipated exogenous event gave us additional leverage to test how increasing the salience of a common enemy interacts with extant partisan tensions. As Fig. 4 shows, Republicans were even less likely to be influenced by Democrats after the assassination than before this event, regardless of which priming condition they received (p < 0.01, N = 485). This finding suggests that Republicans were even less likely to be influenced by the views of Democrats when the salience of the common enemy threat was increased. Our exit survey results are similarly consistent with these behavioral outcomes, since Republicans were found to significantly increase the extent to which they identified with being American during the Iran crisis, whereas no change was observed in Democrats’ degree of national identification amid the crisis (Fig. S8). Our supplementary appendix shows that all of the results above are highly robust to a myriad of statistical tests and methods.

## Discussion

To the best of our knowledge, this study provides the first experimental analysis of the behavioral effects of exposure to a common enemy in the United States during a period of extreme political polarization. Contrary to widespread belief, we found that threats from a common enemy either led to no changes in social learning among partisans or— in the case of Republicans— led their beliefs to be even less influenced by Democrats. These findings are consistent with a growing number of studies that document asymmetric polarization— or patterns of inter-group animosity that appear to be driven more by Republicans than Democrats^22–25^. These results suggest that political narratives about global, combative conflicts—which politicians often invoke to rally patriotic support—may have the unintended consequence of increasing polarization within a nation.

Our study provides several important contributions to the study of political polarization and computational social science more broadly. First, our study contributes to a growing body of work on “backfire effects” in political communication^16,17,23^, where exposure to the attitudes and beliefs of a rival political group have been shown to exacerbate partisan bias. A number of recent studies have found asymmetric backfire effects where partisan bias is particularly amplified among Republicans as a result of cross-party interaction. For example, one recent study of cross-party communication over Twitter found that Republicans were more likely to increase their partisan bias in response to exposure to social media messages from opinion leaders from the opposing party^23^. This result is consistent with social learning experiments which show that partisan priming can lead Republicans to be significantly less cooperative than Democrats when discussing climate change^24^. Since cross-party interaction has been found to consistently entrench partisan bias, a number of studies have proposed that exposure to a common enemy may encourage cross-party influence and cooperation and thereby reduce political tensions^9–14^. However, the results from this study suggest that exposure to a shared enemy may not be sufficient to eliminate partisan boundaries to information sharing and cooperation, and may even amplify political tensions— particularly among Republicans.

More generally, our findings are consistent with the burgeoning theory that in societies that experience extreme polarization such as the United States today, partisan tensions may be high enough that political rivals are perceived as more closely connected to the external enemy than the nation itself^5,7,15–17,19,29,30^. Under such conditions, the threat of a common enemy may increase political tensions among rival groups. Thus, our finding that Republicans learn less from Democrats after exposure to a common enemy may simply reflect their perception that their local opponents are somehow sympathetic to the shared enemy, consistent with recent nationally representative surveys of political attitudes^19^. This sentiment could be observed during the Iran crisis, when many prominent Republican leaders accused Democrats of unduly lamenting the death of a top Iranian general; consistent with our theory, we find that the backfire effects of common enemy priming were stronger after the Iran crisis, and particularly among Republicans, suggesting that the crisis may have intensified Republican perceptions of Democrats as un-American and as threats to the nation comparable to their foreign enemy. Our findings further suggest that exposure to common enemies is especially prone to priming superordinate identities in a divisive way that leads to backfire effects by also evoking the association between one’s rival group and the common enemy, making the rival group into an enemy themselves. In contrast, priming superordinate “American” identity in a more positive manner in our experiment by evoking Fourth of July celebrations did not induce backfiring, consistent with previous work suggesting that positive interventions of this kind are positioned to activate superordinate identities in a non-divisive manner^18^. Our findings therefore add to the growing literature which shows that *how* superordinate identities are primed is critical to whether these identities can unify or divide, with key implications for political media and cross-partisan communication. Together, these findings provide critical insight into the behavioral dynamics of political polarization in highly polarized societies such as the United States.

## Supplementary Information

Supplementary Information.

## Data Availability

All data underlying this study will be made publicly available upon publication, here: https://github.com/eamanj/polarization_paper.

## Code availability

All code underlying this study will be made publicly available upon publication, here: https://github.com/eamanj/polarization_paper.
