# Supplementary material for: An Online experiment during the 2020 US–Iran crisis shows that exposure to common enemies can increase political polarization
Source: Sci Rep. 2022 Nov 11;12:19304. doi: 10.1038/s41598-022-23673-0 (PMC9652360; doi:10.1038/s41598-022-23673-0)
Supplement: Supplementary file 1 — Supplementary Information. [file 41598_2022_23673_MOESM1_ESM.docx]

Supplementary Information for:

**A Randomized Online Experiment during the 2020 US-Iran Crisis shows that Exposure to Common Enemies can Increase Political Polarization**

**This file includes:**

Supplementary Methods

Supplementary Analyses

Supplementary Discussion

Supplementary References

**SUPPLEMENTARY METHODS**

**Recruitment and Full Set of Measures.** We conducted the recruitment for this study using a panel of 3,177 self-identified Democrats and Republicans collected via CloudResearch during spring 2019. Cloud Research (formerly known as TurkPrime) is an online survey company that enables the collection of high-quality survey panels from Amazon Mechanical Turk users using advanced screeners to remove low-quality respondents and inauthentic survey respondents. Though this source does not provide samples that are representative of the United States population, recent research has demonstrated that samples drawn from CloudResearch capture more variation among important demographic and political belief covariates than in-person lab samples (*1,2*). During the profiling process, each of these respondents were asked a series of questions about their demographic characteristics and political beliefs. Between October 2019 and January 2020, we invited 3,162 participants from the online panel to participate in the study. The invitations and study description specified that participants would be compensated $2 for participation with an additional bonus of up to $1 based upon their accuracy in the estimation task (answering the question about U.S. immigration described in the main text of our article). Since we expected performance in the estimation task to depend on the feedback participants received from the bots—and since bot feedback in the first round was based on initial participant input (participant input +/- 50 percentage points)—we ultimately compensated all respondents with the full bonus amount regardless of the accuracy of their answers.

1,692 out of the 3,162 people invited to join the study initially agreed to participate for a raw response rate of 53.5%. As Table S1 below shows, we observed no significant differences in response rates between Republicans and Democrats. We ran additional models (not shown) that examined whether age, gender, race and ethnicity, level of education, or income level were significant predictors of non-response. Of these, only age was a significant predictor of non-response: older people were slightly more likely to participate (*p* < 0.001). Our sample also compares favorably to the national population on each of these measures. The mean age of respondents in our sample is 42.2 compared to the national average of 37.84 reported by the 2016 American Community Survey. Our respondents were 52.2% female, compared to the national average of 51%. Our sample contained fewer racial minorities than the national average, however. Our sample was 82.2% white or caucasion compared to 70% (the national average).


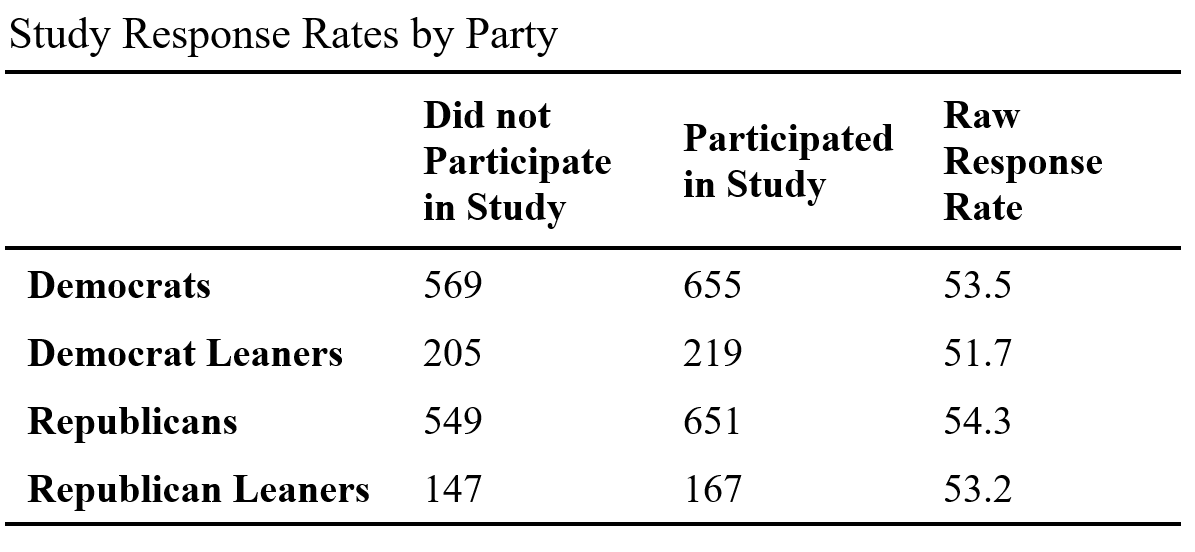


**Table S1:** Study Response Rate by Party Identification.

Before they were forwarded to the online platform where they performed the collaborative task with a bot impersonating a member of the opposing party, respondents were sent a link that redirected them towards an interface that asked them to provide their MTurk “worker id” (a unique identifier given to each Mturk worker by Amazon). On that platform, participants first read an informed consent dialogue detailing the study process, the conditions for compensation, and their rights as study participants. If they consented to participate in the study, respondents were then directed to the study’s main online platform where they first were shown a profile populated with the age, gender, and party identification they listed for themselves when they first joined the panel used to recruit respondents to the study. Participants were offered the opportunity to change these details before proceeding to the next stage which introduced them to the estimation task.

Before entering the priming conditions, subjects participated in an example of the estimation task to ensure they understood the instructions. During this example task, subjects were shown the experimental estimation interface (figure 1 in the main text of our article), but with a different political question: “What percentage of food stamp (SNAP) recipients do you believe are employed full-time or part-time?” After completing the example estimation task, participants were randomized into one of three priming conditions (the neutral, patriotic, or common-enemy prime) conditions. Subjects were presented with a prompt stating: “Please read the following article taken from Reuters, a non-partisan news outlet. On the next screen, we are going to ask you several questions about the article. You will need to answer those correctly to advance to the game.” Next we asked respondents to answer three questions about facts presented in the article. Subjects were able to retry answering these questions until they answered each of them correctly. Once each question was answered correctly, subjects entered the estimation task, where they were asked to provide an estimate to the following political question described in the main text of our article: “Thinking of all new immigrants to the U.S. between 2011 and 2015, that is all individuals who arrived in the U.S. between 2011 and 2015, but were not U.S. citizens at birth, what percentage were university-educated?” At Round One, all subjects regardless of condition provided an independent estimate, without exposure to the estimate from the bot impersonating a member of the opposing party.

At round two of the collaborative online task on our platform, subjects were given an opportunity to revise their estimate after being exposed to the estimate of a bot described as a member of the opposing party. This bot was programmed to initially provide an estimate that was exactly 50 percentage-points away from the participant’s estimate. This manipulation allowed us to test the effect of each priming condition on the willingness of participants to revise their estimates toward the estimate provided by the bot. For each bot guess after round two, the bot either stuck close to its initial guess (the stubborn bot) or moved its guess closer to the participant’s guess (the friendly bot updated in a memoryless Bayesian fashion, such that the prediction for bot in round $t+1$is $x_{bot,t+1}=\frac{3}{4} x_{bot,t}+\frac{1}{4} x_{participant,t}+Uni(-3, 3)$). Participants were randomly assigned to interact with the stubborn or friendly bot for all rounds of the game.

After completing five rounds of the game, participants completed an exit survey with several measures in the following order. First, participants were asked a political knowledge question: “Which party has a majority of seats in the Senate?” Next, they were asked to identify the political party of the other player in the game as a manipulation check. They then estimated the political knowledge of the other person (i.e. the bot) in the game. Participants indicated their level of identification with five different groups using a single-item measure of identification: Americans, people in their state, people in their neighborhood, Democrats, and Republicans (*3*). They completed a mood measure (the PANAS), and feeling thermometers towards ingroups (their political party, and all americans, and outgroups (the opposing political party, Russians, Iranians, and Chinese), and two distractor groups (Texans and Californians) (*3*). Finally, participants responded to several questions about their experience in the game, and were debriefed and compensated for their time. A video that illustrates the entire participant experience is available [online](https://drive.google.com/file/d/1mKvdEi56UndpI8yTfhDuCOiB1P6oR1aP/view?usp=sharing).

**Selection of News Articles for Treatment Conditions.** In order to identify neutral, patriotic, and common-enemy primes, we conducted multiple rounds of pre-testing of news articles to ensure that each one created a) no response; b) a prideful response; or c) a fearful or threat response. Our pre-testing effort was further designed to ensure that neither Democrats nor Republicans were significantly more likely to respond to one of these primes. We chose to select articles from Reuters because previous studies indicate it as the most centrist news organization at the time of this writing (*4*). We gathered and pre-tested a total of 42 articles from Reuters (12 neutral articles, 12 articles that prime patriotism, and 18 that describe a common enemy). To ensure comparable length — and to expunge explicit partisan cues from the text of the prime — we edited the original Reuters articles before pre-testing them to an average length of 400 words. Each article was associated with a single image, sourced from the original publication.

Between March 29th and April 1st, 2019 we pretested the 42 articles by asking 566 respondents (347 Democrat and 219 Republicans) recruited from MTurk, to complete a survey on Qualtrics about their attitudes and feelings. This survey presented respondents with one article, randomly selected out of the set of 42 total articles, and asked the same set of questions used in the exit survey of the main study. We used an iterative process to select the three final articles. First, we searched for the article in each category (neutral, patriotic, or common enemy) that created equal shifts in party identification, identification with America, and emotional responses among both Republicans and Democrats. To ensure that respondents viewed the potential primes as realistic news stories, our pre-testing survey also included open-ended questions designed to determine whether respondents suspected the articles had been edited or fabricated. We received no indications from respondents that they expected such editing or manipulation.

All of the articles used as primes in this experiment are provided below. The prime for the control condition is shown as fig. S1, for the patriotic condition as fig. S2, and for the common enemy condition as fig. S3. Our analysis suggested the best “neutral” prime was an article titled “Stone in African cave boasts oldest-known human drawing” (Table S2). The article focused on details of an archaeological excavation in Blombos Cave, near the Indian ocean. To select the ingroup prime, for each candidate article, we compared self-reported importance of being American to MTurkers after reading the article with the levels recorded among respondents randomly selected to read the candidate neutral article. We found only one article that effectively primed patriotism, by generating a significant difference in self-reported American identity among Republicans and Democrats (*n*=173, p>|z|=0.073, Wilcoxon rank sum test, Table S3). The article was entitled “America celebrates July 4th with hot dogs, banners and barbecues,” and discussed Fourth of July celebrations in various sites throughout the U.S., including both cities with a large population of Democrats (e.g. Austin, Texas) and Republicans (e.g. Gainesville, Florida). A similar article was used to prime patriotic responses in a previous study (*5*).

Selecting the article that created an equally strong sense of threat or fear among both Republicans and Democrats proved more challenging. In our initial round of testing, we discovered no suitable article— though the issue was mostly related to the *amount* of threat experienced by respondents, not the consistency of this feeling across members of both parties. This led us to select four articles for a second round of pre-testing with an expanded sample on Qualtrics as well as a small sample on the main study platform. We used this second round of pre-testing to get more precise estimates of the identification measures described above and to assess the viability of the articles in the context of the experimental platform. To ensure that our respondents would feel sufficiently threatened, we also created and tested a new common enemy prime by combining content from three news stories from Reuters about possible threats from Iran, China, and Russia into a single article entitled “U.S.-Iran tensions rise among downing of U.S. Military Drone, China, Russia affirm Tehran’s right to sovereignty.” By combining real-world news stories, the article discusses a scenario where Iran has shot down a U.S. drone, thereby raising fears of a military confrontation; meanwhile, the article suggests that China and Russia both independently sided with Iran in the conflict, thus suggesting the growing threat of a joined threat from all three enemies of the U.S. We found that this article significantly primed American identity compared to the neutral article (Table S4). Therefore, we selected it as our common enemy prime.

**
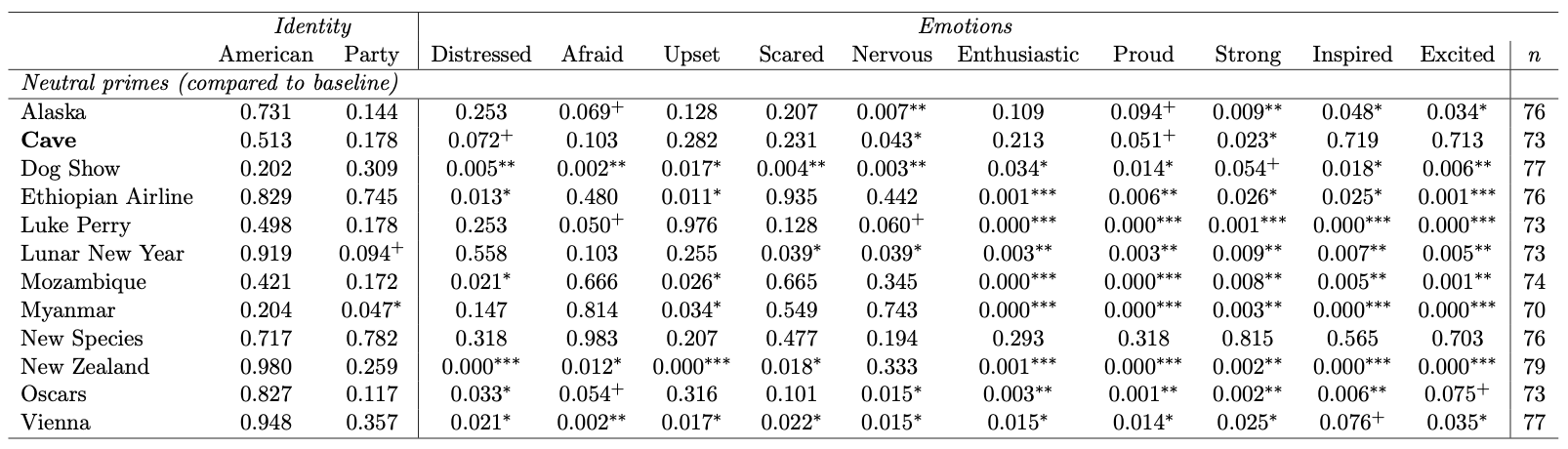
**

**Table S2. Wilcoxon Rank Sum Tests for Identity and Emotions scores for candidate neutral articles, compared to a baseline. Selected article in bold.**


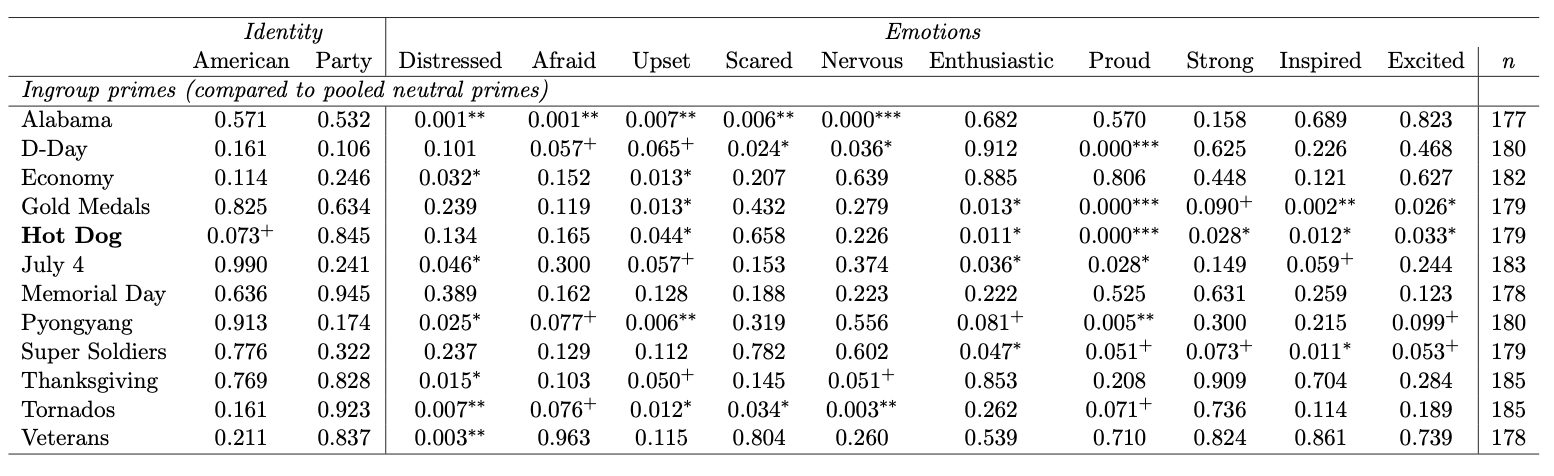


**Table S3. Wilcoxon Rank Sum Tests for Identity and Emotions scores for patriotic, compared to neutral articles. Selected article in bold.**

**
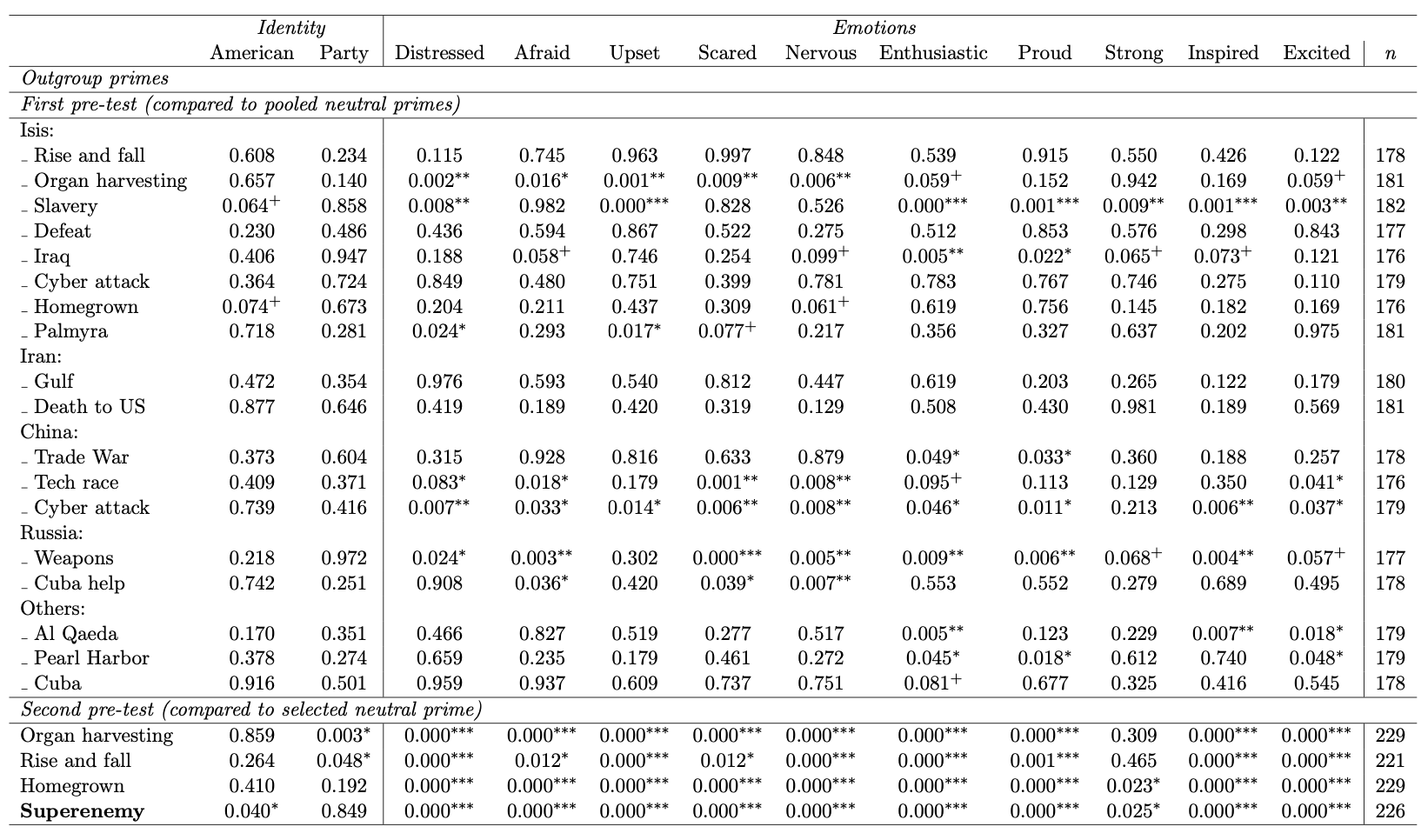
**

**Table S4. Wilcoxon Rank Sum Tests for Identity and Emotions scores for outgroup articles. Selected article in bold.**


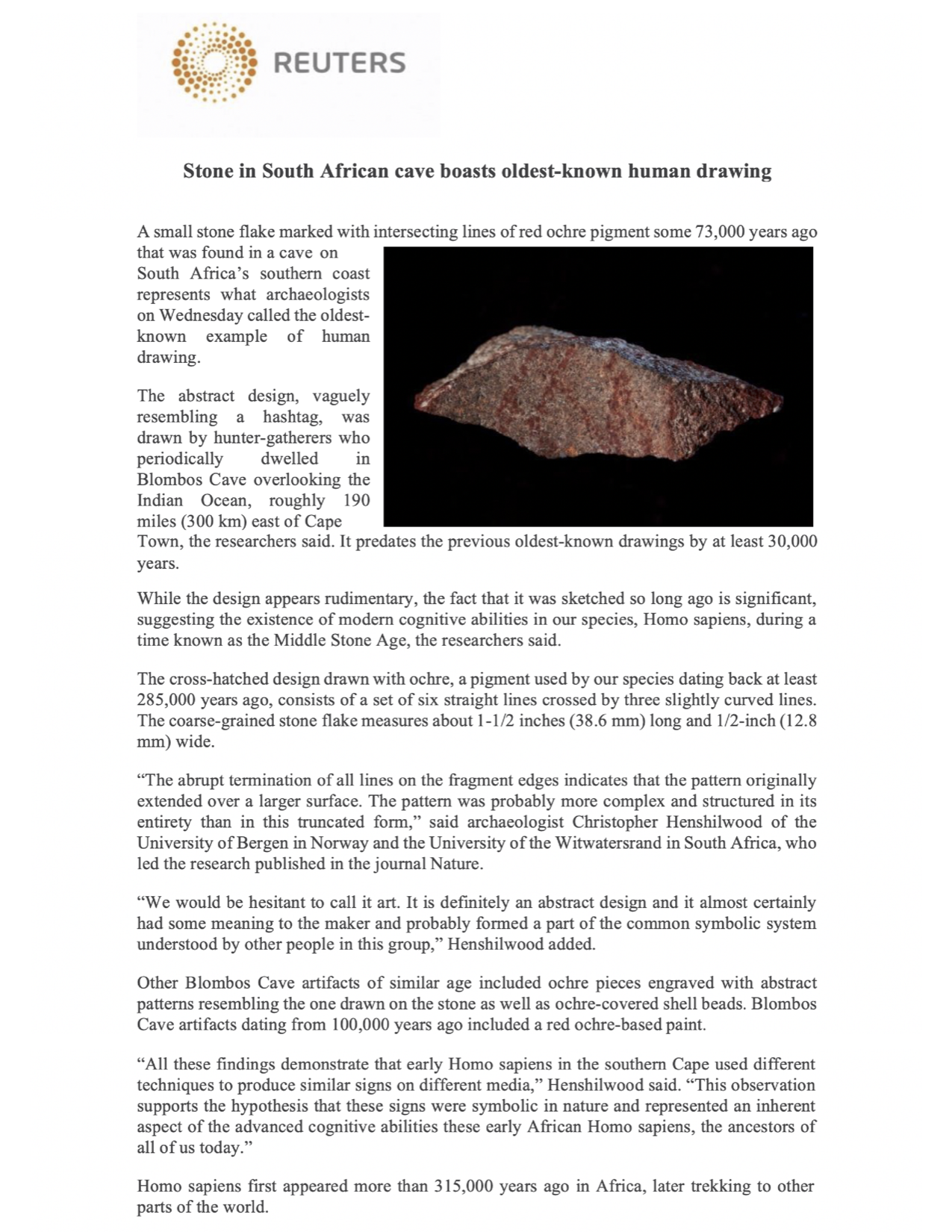


**Figure S1**: The article used in the control condition.


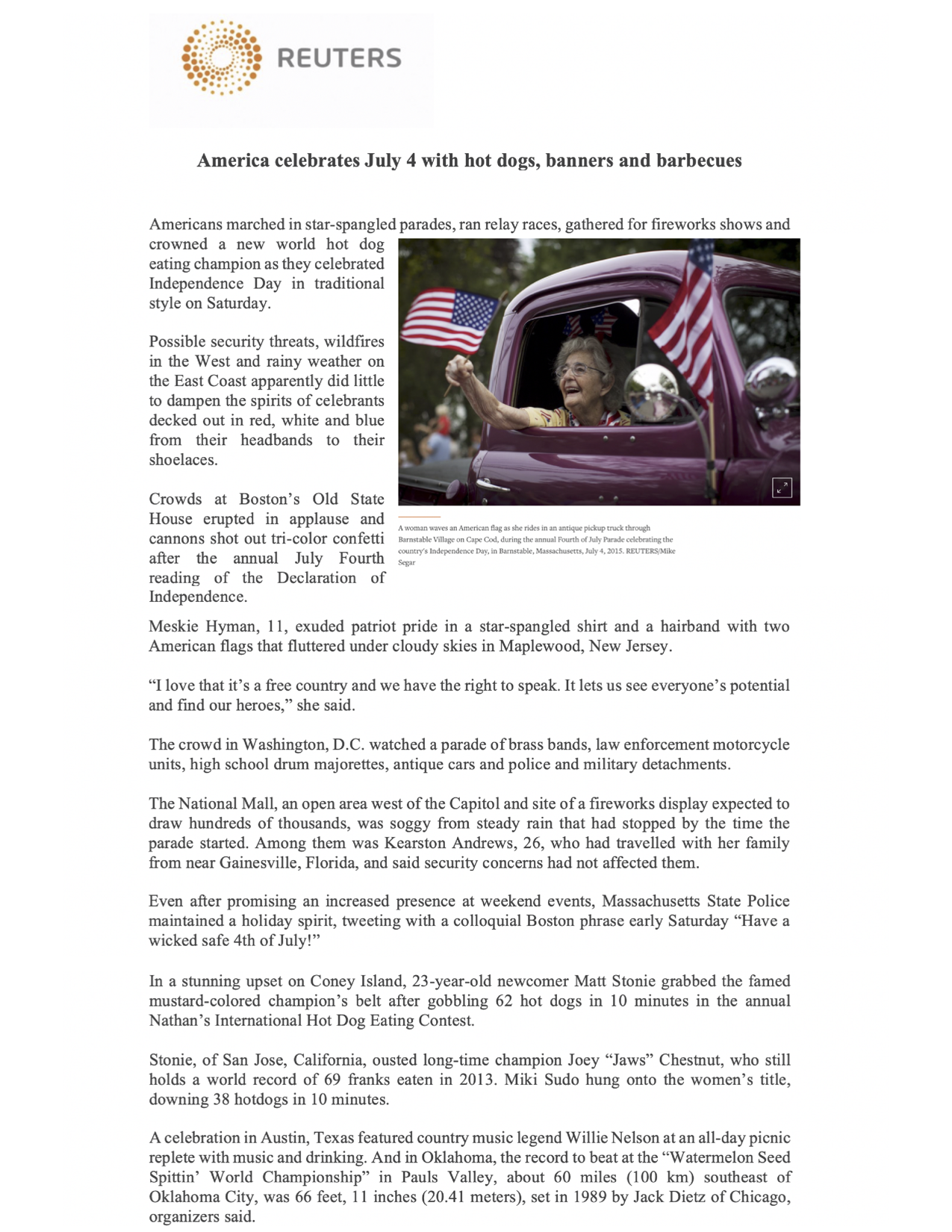


**Figure S2:** The article used in the patriotic priming condition.


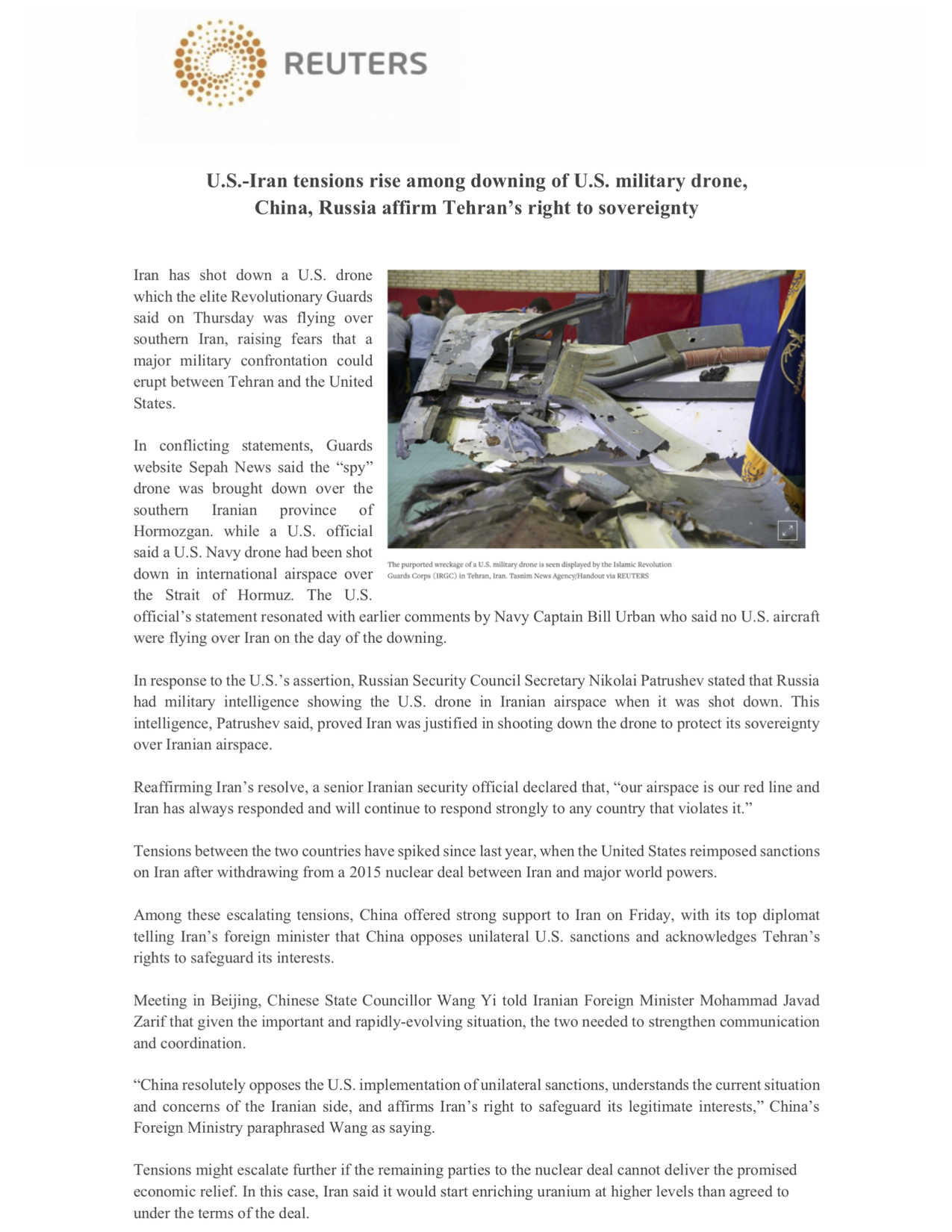


**Figure S3:** Article Viewed by Respondents in the Common Enemy Priming Condition

**Post-Stratification Modeling Strategy.** In this section we explain the methodology we used to obtain results in figures 2, 3 and 4. In both post-stratification and other robustness models, the dependent variable is the amount the participants updated their estimates in response to the estimate made by the bot impersonating a member of the opposing party. If the participant updates their first estimate to move closer to the bot's estimate, the dependent variable is positive. In contrast, if the participant moves away from the bot, the dependent variable is negative. The equation below formalizes the definition of our dependent variable:


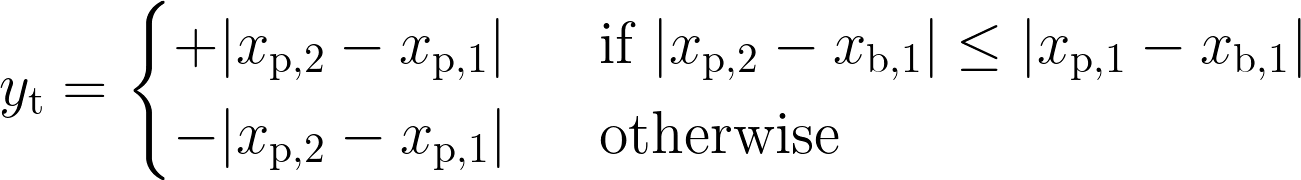


where *y* is the dependent variable corresponding to the update and *x_p,1_ , x_p,2_, x_b,1_* correspond to the player's first and second estimates and bot’s first estimate respectively.

To generate our main results, we estimated the mean update toward the bot, accounting for the variation in composition of participants by gender, political knowledge, their initial estimate and whether they correctly identified the political affiliation of the other user on the platform within each prime condition. Effectively, we treated the full sample of participants in our study as the reference population and used post-stratification to compute mean updates that have controlled for variation in strata size across treatments.

Different strata were defined for each possible combination of covariates mentioned above. Next, we discuss the rationale for controlling for each of these variables. A key assumption of our research design is that respondents realize they are collaborating with a member of the other party during the estimation task in our online platform. Though we used strong visual cues to communicate this to respondents, our exit-survey also included a question that asked respondents whether they remembered the party affiliation of the other user on the online platform. Hence, our post-stratification analysis used a binary indicator of whether respondents correctly identified the political affiliation of the other user (i.e. the bot) on the platform, as one dimension of each strata definition.

Because the initial estimates of our bots were programmed to always be 50 percentage points plus a small random noise away from our respondents (on a 0-100 scale), this response may be more or less credible depending upon the respondent’s initial guess. For example, respondents who guessed that only 10% of recent immigrants hold a college degree would view an estimate of 60% from the bot, which would be more plausible than another respondent who guessed 48%, which would provoke a guess of 98% from the bot. To adjust for the initial estimates, we converted respondents' first estimates to categorical variables by binning them into intervals of length 25, and used this four-level categorical variable as another dimension of strata definition.

The exit survey included a question about which party has a majority of seats in the Senate. This question was used to test whether the respondent had high political knowledge. Our post-stratification estimate thus includes a binary indicator of whether the respondents correctly answered this question as another factor in strata definition. Finally, there is some evidence in the literature on the existence of the gender gap in political partisanship (*6*). Therefore, we control for the gender of the respondent as a binary variable in our analysis.

The equation below shows how the mean update within each prime treatment can be estimated using post-stratification:


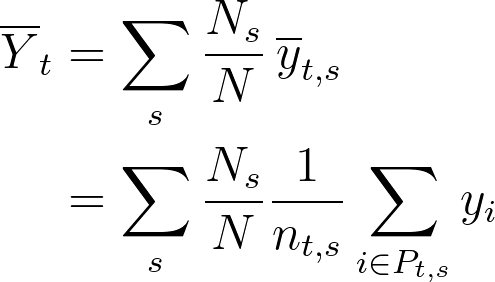


where $\underline{Y_{t}}$ is the post-stratified estimate of mean update in prime treatment *t*, *N_s_* is the number of participants in strata *s* across all treatments, *N* is the total number of participants across all primes treatments. *y_t,s_* is the average update of participants in prime treatment *t* and strata *s*. *P_t,s_* and *n_t,s_* are the set and size of such participants respectively, and finally *y_i_* is the amount participant *i* updated their initial estimate after observing the bot’s estimate. As noted above, the estimate $\underline{Y_{t}}$ treats all recruited participants as the reference population and weighs the average of each strata based on frequency of that strata in the population.



The point estimates from the equation above are shown in figure 2. The point estimates shown in figures 3and 4are computed in a similar fashion with the difference that *t* would correspond to level of ingroup favoritism or timing with respect to the assassination. The variance of $\underline{Y_{t}}$ can be estimated using equation below (*7*):

[
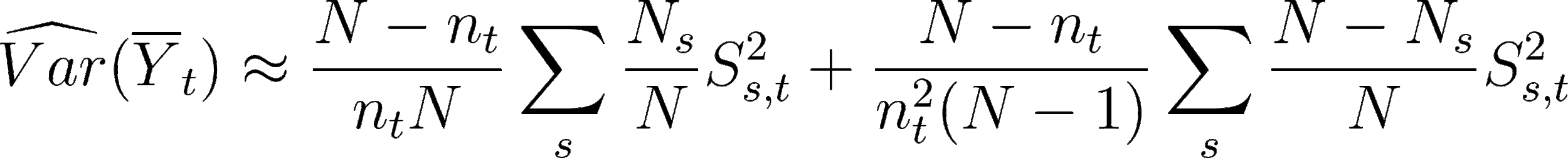
](https://latex-staging.easygenerator.com/eqneditor/editor.php?latex=%20%5Cwidehat%7BVar%7D(%5Coverline%7BY%7D_t)%20%26%5Capprox%20%5Cfrac%7BN%20-%20n_t%7D%7Bn_t%20N%7D%20%5Csum_%7Bs%7D%20%5Cfrac%7BN_s%7D%7BN%7D%20S_%7Bs%2Ct%7D%5E2%20%2B%20%5Cfrac%7BN%20-%20n_t%7D%7Bn_t%5E2%20(N%20-1)%7D%20%5Csum_%7Bs%7D%20%5Cfrac%7BN%20-%20N_s%7D%7BN%7D%20S_%7Bs%2Ct%7D%5E2%20#0)



where *n_t_* is the number of participants in treatment *t* and $S_{s,t}^{2}$ is the sample variance of updates in prime treatment *t* and strata *s*. Given the estimate of variance above, we can construct confidence intervals as shown in figures 2, 3, and 4 in the main text. We can further perform post-stratification adjusted t-tests to compare mean updates between various conditions. We used the R package survey to perform the two-sample t-tests between treatments (*7*).

To produce the results in figure 3 of the main text, we created a measure that describes how much Republicans identify with their own party or their ingroup bias. More specifically, we calculated the difference between each respondent's ingroup and out-group feeling thermometer. We then labeled those participants in the top 10th percentile of this measure as strongly partisan with high ingroup bias and the remaining individuals as moderate partisans. An alternative method to measure the strength of partisanship is to use the raw measure of feeling toward the ingroup party. We can then treat the participants in the top 10th percentile of this measure as strongly partisan and the rest as moderately partisan. The 10th percentile cutoff for Republican participants in our data corresponds to 99 out of 100 percentage points for feeling toward Republicans. We use this alternative measure as a robustness check to our result based on the normalized feeling thermometer measure analyzed in the main text. Figure S4 shows the post-stratified difference between these two groups of Republicans.

Fig. S4 The extent that Republicans updated their beliefs toward the opinion of the Democrat bot across all prime conditions with a bot impersonating a Democrat during the online task by an alternative measure for strength of partisanship. Vertical axis describes the post-stratified average belief update (in percentage points), where strata are defined by gender, political knowledge, the accuracy of initial guess, and awareness of bot’s membership in the opposing party. Strong republicans are defined as those who are in the top 10th percentile of an ingroup feeling thermometer measure. Error bars display 95% confidence intervals. * p < 0.05.


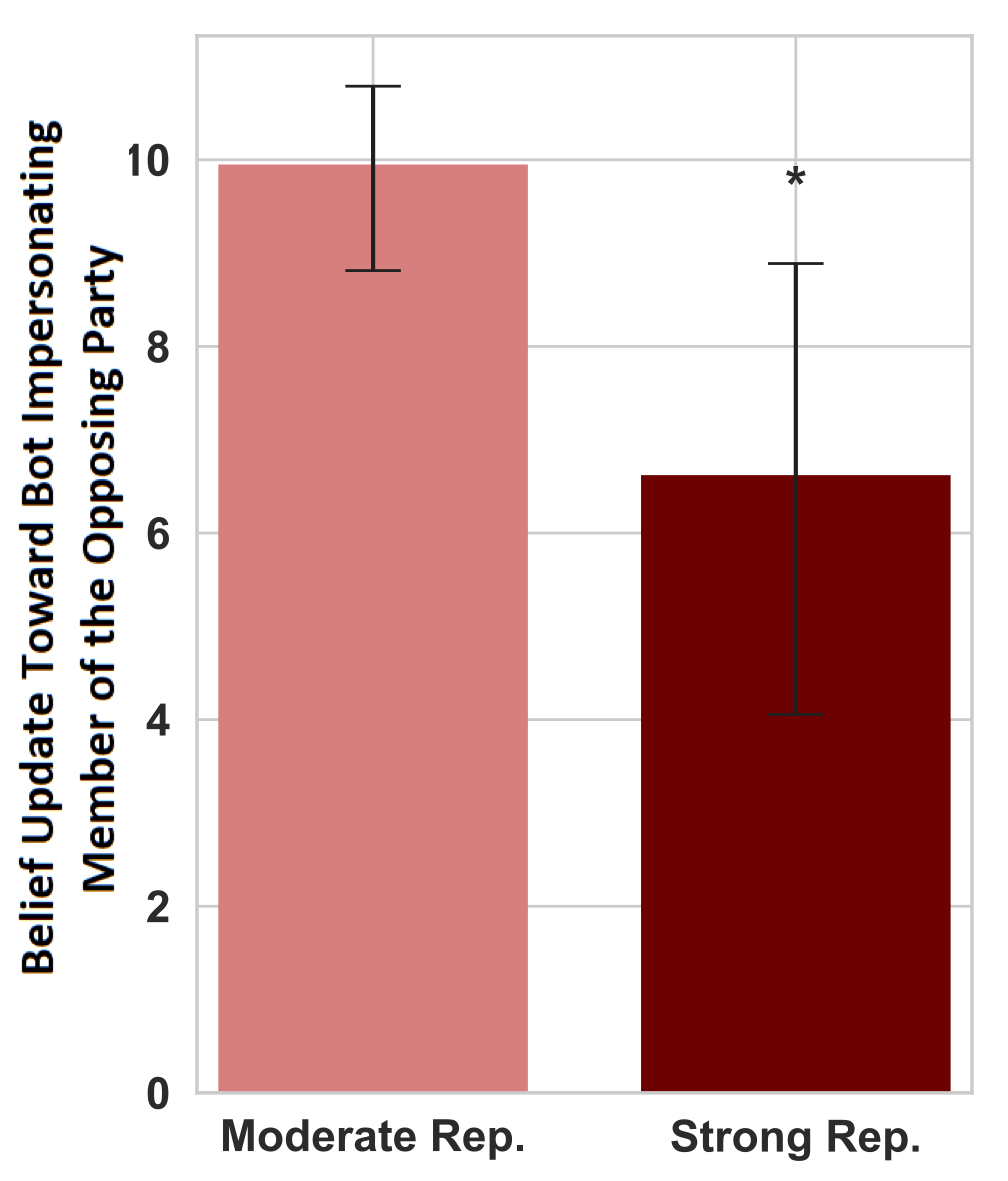


**SUPPLEMENTARY ANALYSES**

**Full Results from Models and Robustness Checks.** In this section we report full results of the models summarized in the main text of our paper as well as a series of robustness checks for Republicans and Democrats.


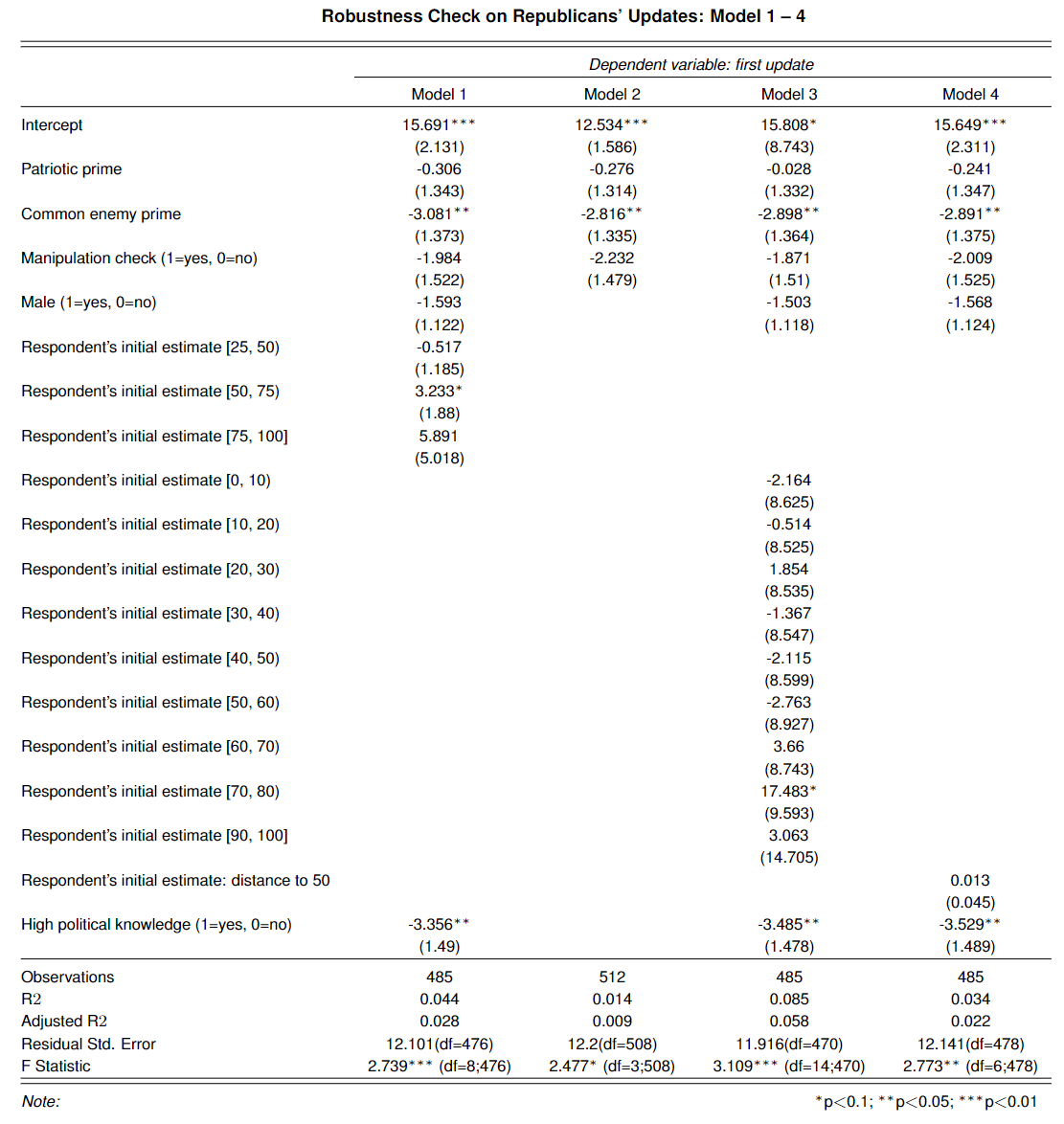


**Table S5.** Robustness check of Republicans’ belief updates toward the opposing party bot across experimental conditions. Models 1 to 4.


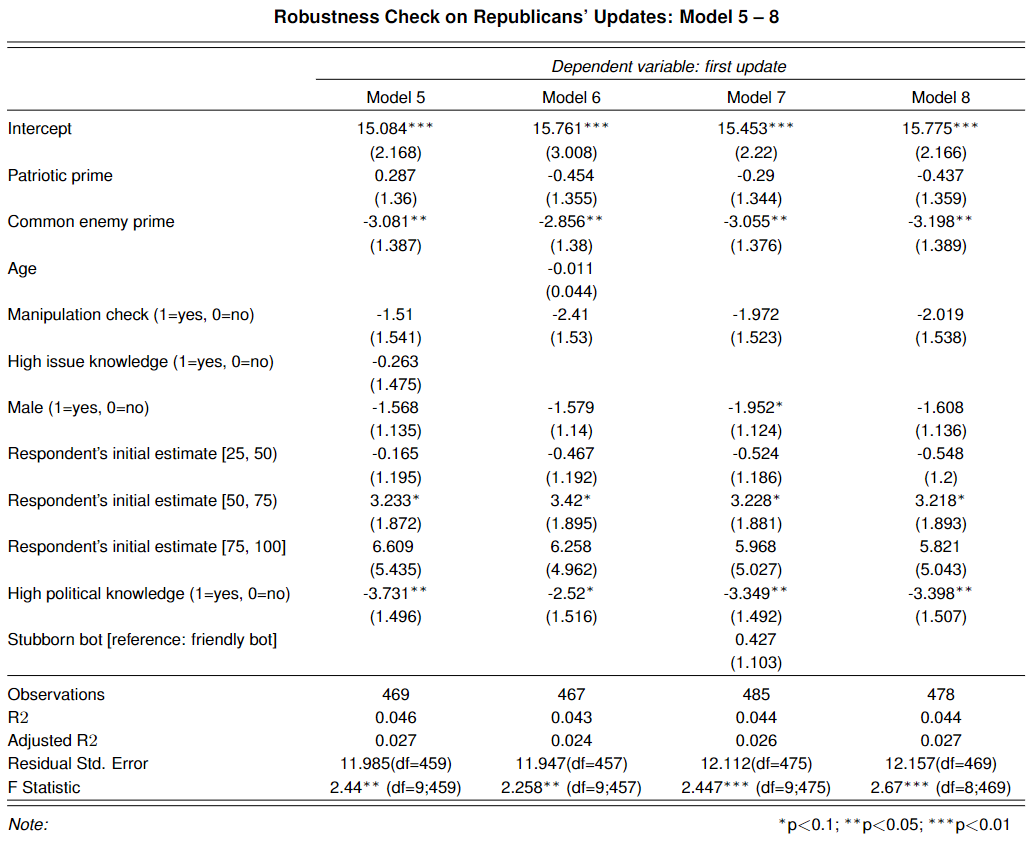
**Table S6.** Robustness check of Republicans’ belief updates toward the opposing party bot across experimental conditions. Models 5 to 8.

**
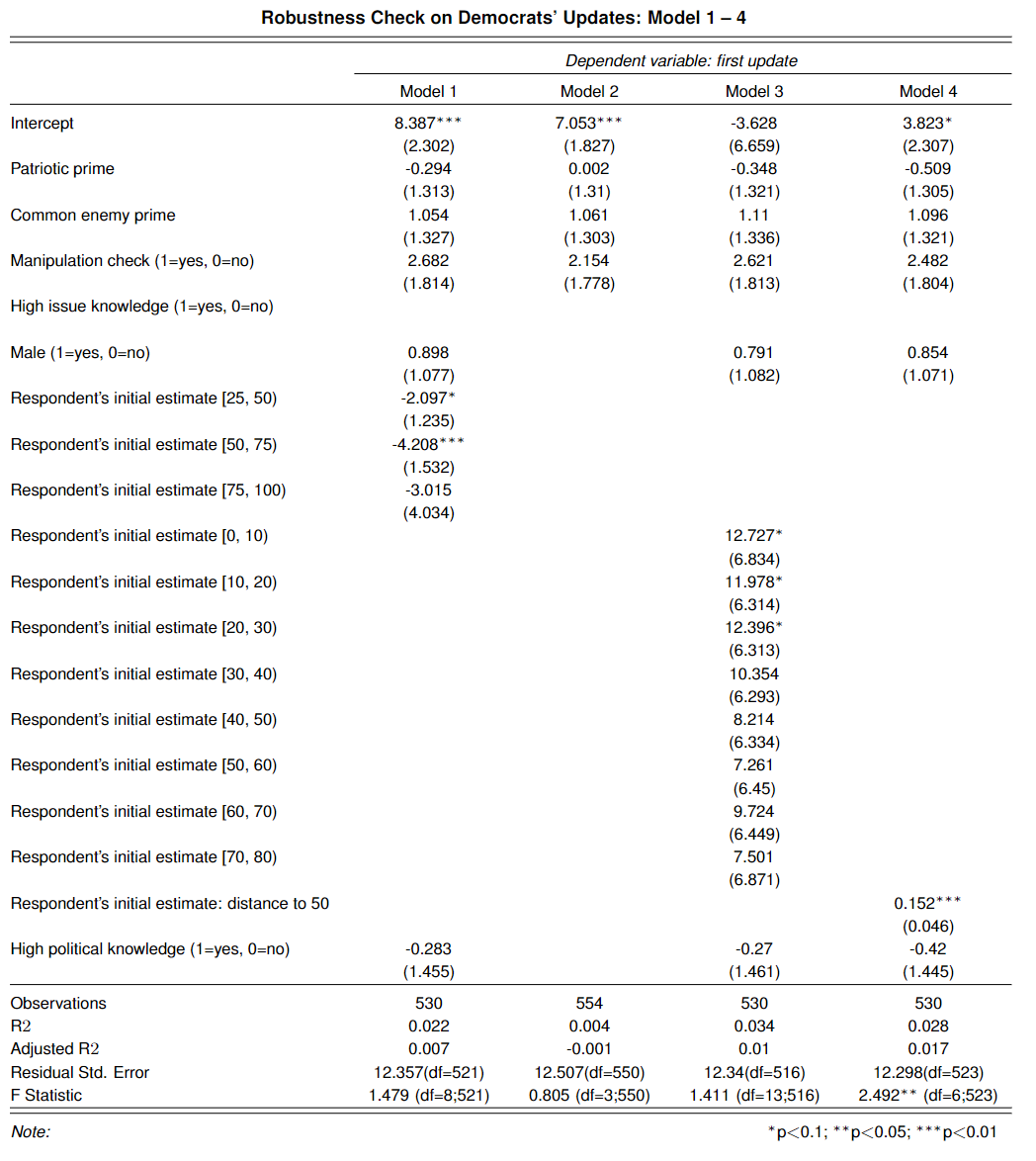
**

**Table S7.** Robustness check of Democrats’ belief updates toward the opposing party bot across experimental conditions. Models 1 to 4.


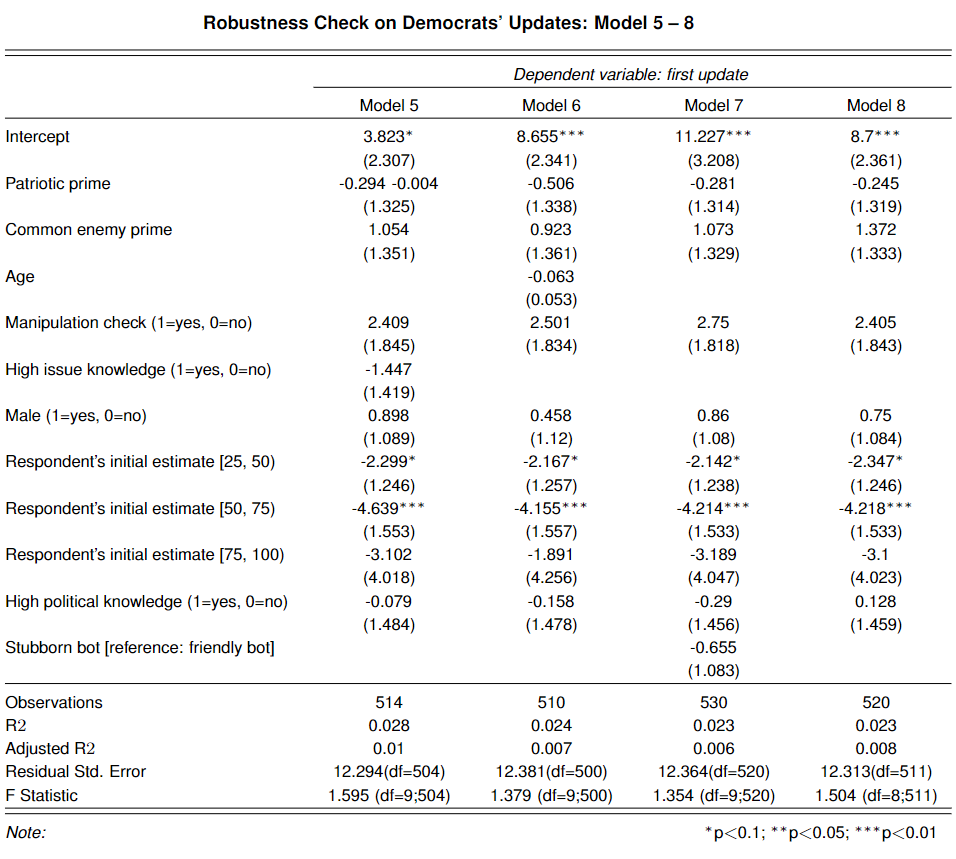


**Table S8.** Robustness check of Democrats’ belief updates toward the opposing party bot across experimental conditions. Models 5 to 8.

**Model 1: Full Results from Figure 2 in Main Text.** Model 1 reports the full results of the first two models of updating behavior among Republicans (Table S5) and Democrats (Table S7). This model includes binary indicators that describe the patriotic and common enemy primes. A key assumption of our research design is that respondents realize they are collaborating with a member of the other party during the estimation task in our online platform. Though we used strong visual primes to communicate this to respondents, our exit-survey also included a question that asked respondents whether they remembered the party affiliation of the other user on the online platform. Our models thus include a binary indicator of whether respondents correctly identified the political affiliation of the other user on the platform.

**Model 2: Sensitivity of Results to Respondent’s Initial Estimate.** Model 2 assesses the sensitivity of our findings to heterogeneity in the initial estimates provided to the question task on our online platform (Republicans, Table S5; Democrats, Table S7). Because the initial estimates of our bots were programmed to always be 50 points away from our respondents (on a 0-100 scale), this response may be more or less credible depending upon the bot’s initial guess. For example, respondents who guessed that only 10% of recent immigrants hold a college degree would view an estimate of 60% from the bot, which would be more plausible than another respondent who guessed 48%, which would provoke a guess of 98% from our bots. In order to assess the sensitivity of our results to such bot behavior, we binned respondents according to their initial estimate range and reported these categorical measures in Model 2 in Tables S5 and S7. As these models show, the effects discussed in the main text of our article remain despite the inclusion of these additional indicators in this model.

**Model 3: Pre-Existing Knowledge of Issue in Estimation Task.** In our study’s exit-survey, we asked whether the players are familiar with the issue in the estimation task (immigration). We collected this measure in order to determine whether people with more knowledge about the issue may be less likely to update their estimates in response to the bot regardless of priming condition or political party. Model 3 in tables S5 and S7 adds a binary indicator of familiarity with the issue, showing that the inclusion of this additional indicator does not substantively alter the results we report in the main text of our article.

**Model 4: Age Effects.** There is some evidence in the scholarly literature on political polarization that political beliefs become more resistant to change across the life course. For this reason, we included a continuous measure of respondent age that we report in Model 4 in Tables S5 and S7. Once again, the results we report in the main text of our article hold despite the inclusion of this additional indicator.

**Model 5: Variation in Bot Behavior.** In order to capture real-world variation in how members of opposing parties might interact with each other on our platform, our study randomized respondents to perform the estimation with a “friendly” bot or a “stubborn bot.” While the former always updated towards the respondent’s estimate, the latter held fast to its own initial estimate. To examine whether the behavior of the bot interacts with our treatment effects, Model 5 in tables S6 and S8 reports interactions between the type of bot each respondent interacted with and each prime. We observed no significant effects of this interaction for any of our models and the main effects of the common enemy prime remain consistent among Republicans, as before.

**Model 6: Strength of Partisanship.** Model 6 reports the full model reported in figure 3 in the main text of our article, which analyzes heterogeneous treatment effects according to the strength of partisanship among Republicans and Democrats. We measured the strength of partisanship using the feeling thermometer measures we collected in our study’s exit-survey. The binary indicator reported in Model 6 describes respondents whose feelings towards their own party are in the top 10% of favorability ratings (on a 0-100 point scale). As Tables S6 and S8 show, we observe significant negative updating behavior (i.e., less cooperation with the bot impersonating a member of the opposing party) among Republicans, but not Democrats. This effect is robust to other cut-off points, though the effect becomes weaker when strong partisans are defined as those in the top 20 or 30% of respondents (in terms of strength of affection towards their own party).

**Model 7: Inconsistent Reporting of Party Identification.** Because we collected respondents’ political identification from the panel data originally used to identify respondents as well as on the platform itself, we were able to identify respondents who inconsistently reported their party identification. Though it is possible that some respondents changed political parties in the interim period, it is considerably more likely that these respondents were either responding expressively or were not paying attention to the online platform’s onboarding questions. As an additional robustness check, Model 7 in Tables S6 and S8 removes these respondents from the model, producing results that are nearly identical to those we report in the main text of our article.

**Model 8:** Main controls, where we keep the last position of the slider if the player did not enter for the second round (Republicans, Table S6; Democrats, Table S8). For those players who do not enter the second round, we use the last position of the slider as their responses for 64 Republicans and 66 Democrats. After we add these individuals, the effect of enemy priming on the republicans disappears.

**Updating Behavior Beyond the First Round.** Thus far we have only discussed the updating behavior of respondents after they viewed the estimate of the bot impersonating a member of the opposing party for the first time. This is because the most consequential effect of exposure to a member of the opposing party is likely to occur during the initial exposure. Yet our study allowed respondents to update their estimates during two subsequent rounds of updating in response to the bot’s estimates. Even though exposure to party affiliation during these rounds is redundant, we analyzed updating behavior among Republicans and Democrats across each of these rounds but observed negligible or small treatment effects in so doing. Between the second and third round of updating, we observed no significant differences in the updating behavior of Republican respondents (p > 0.05, N = 464) and a small negative updating effect among Democrats who were exposed to the common enemy prime (p < 0.05, N= 504) but not the patriotic prime (p > 0.05, N = 504). Between the third and fourth round of updating, we observed no statistically significant differences in updating behavior among either Republicans (p > 0.05, N = 441) or Democrats (p > 0.05, N = 487).

**Robustness check for the findings about strong partisans.** As an additional robustness check for the findings reported in figure 3, we employ regression models with variations on the number of bins. Similar to the results we reported about the effect of partisanship using two different measures of party identification in figures 3 and S4, Table S9 below also shows that the effect of partisanship is consistently negative and significant for our two different measures of ingroup bias. The effect is stronger with 10 bins in the initial estimate than 4 bins, however.


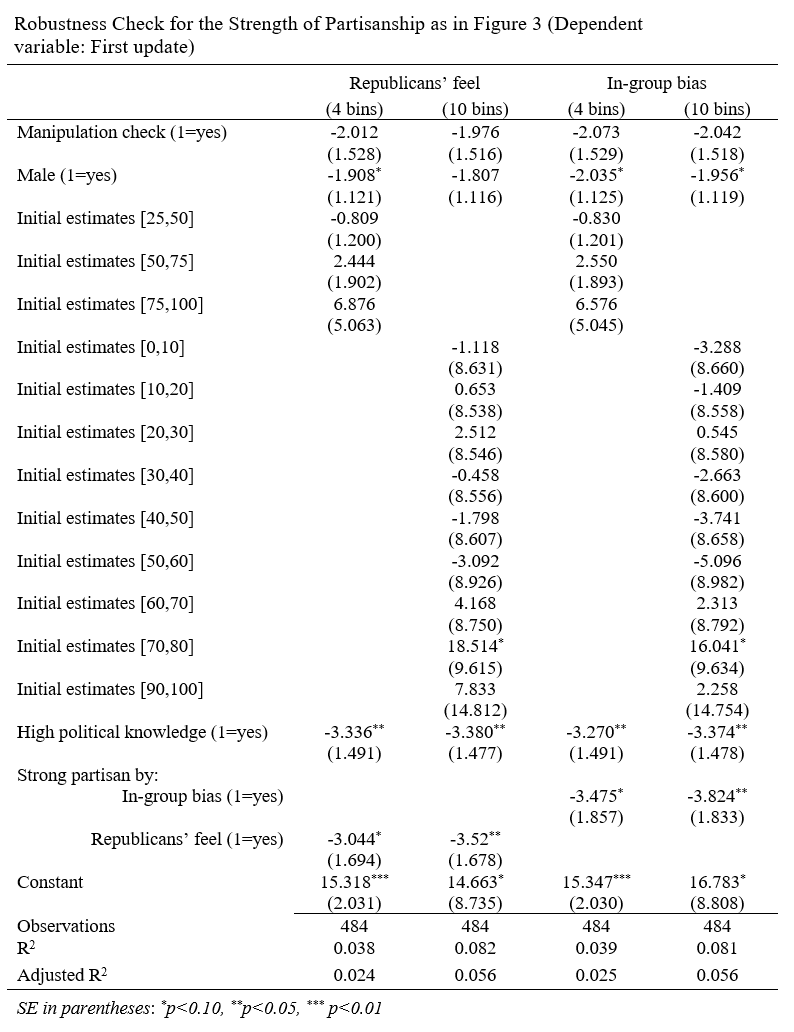


**Table S9**. Robustness check for the strength of partisanship as in figure 3.

**Robustness check for the effect of the Iran crisis.** Table S10 below presents a robustness check on the results reported in figure 4. The binary indicator reported in Table S10 is an indicator for whether the experiment is conducted before or after the Iran Crisis. As Table S10 shows, we observe Republicans were significantly less likely to cooperate with Democrats after the assassination than before this event with 10 bins. The effect is weaker with 4 bins, however.


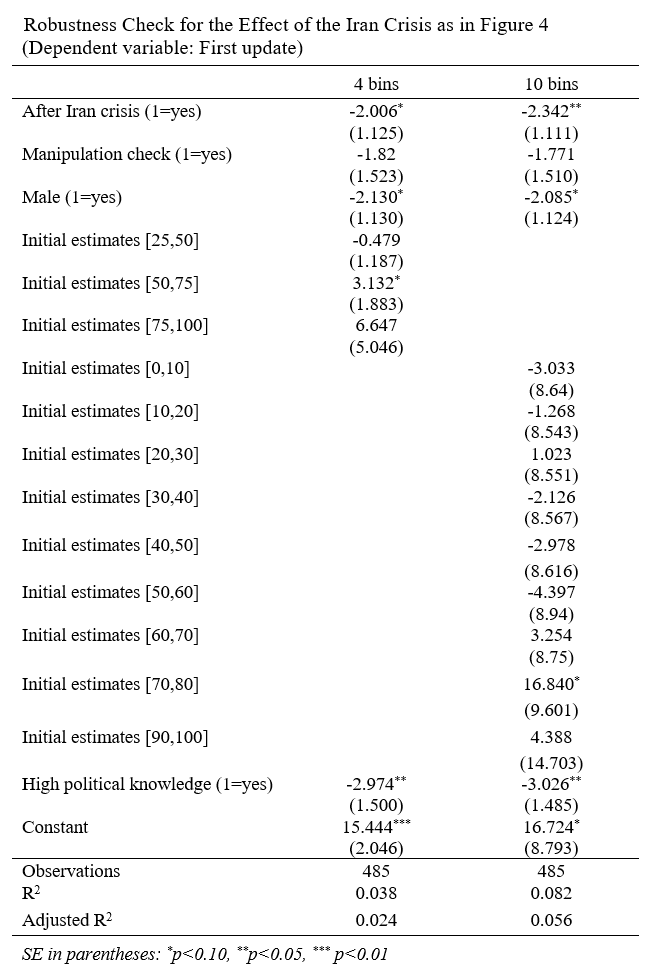


**Table S10**. Robustness check for the effect of the Iran crisis as shown in Figure 4.

**Supporting our Mechanism via Exit Survey Results**. Immediately after the main experiment, participants from all conditions were invited to complete an exit survey, in which they were asked to indicate the extent to which they identified with (i) being American, with (ii) Republicans, and with (iii) Democrats. Specifically, they were asked to answer the following questions.

How strongly do you agree or disagree with the following statements?

*-I identify with Americans*

Options: Strongly Disagree, Disagree, Neither Disagree or Agree, Agree, Strongly Agree

*-I identify with Democrats*

Options: Strongly Disagree, Disagree, Neither Disagree or Agree, Agree, Strongly Agree

*-I identify with Republicans*

Options: Strongly Disagree, Disagree, Neither Disagree or Agree, Agree, Strongly Agree

These exit survey questions provide a supplementary set of outcome measures that allow us to provide additional evidence for our proposed mechanism. This proposed mechanism suggests that the common enemy article primes American identity, but that due to polarization, Republicans and Democrats differ in their representation of American identity, which is exclusive toward each other. Specifically, we predict that Republicans will be especially prone toward a view of American identity that increases, rather than decreases, receptivity toward and inclusion of Democrats, given a recent nationally representative survey which shows that Republicans are significantly more likely to view Democrats as un-American and as threats to the nation, compared to Democrats’ attitudes toward Republicans (*8*). These survey measures allow us to validate the plausibility of this mechanism by measuring (i) whether the common enemy article primed American identity, (ii) whether this activation of American identity was similar or different among Republicans and Democrats, and (iii) whether this activation of American identity translated into the inclusion or exclusion of increased identification with the other party.

First, fig. S5 shows that across all conditions Republicans identified with being “American” significantly more strongly than Democrats (N=893, *p*<0.0001, χ2 test), consistent with prior research showing that Republicans exhibit higher overall levels of patriotism (*9,10*). Next, fig. S6*A* shows that Republicans identified significantly more strongly with being American after reading the common enemy article as compared to Republicans who read the neutral control article (N=274, *p*=0.05, χ2 test). Yet, fig. S6*B* shows that this increase in Republicans’ identification with being American did not co-occur with an increase in the extent to which they identified with Democrats (N=274, *p*=0.62, χ2 test); instead, Republicans appear to have increased their sense of national identity in an exclusive manner that did not include increased cross-party identification. Meanwhile, fig. S7 shows that Democrats were significantly less likely to identify with being American after reading the common enemy article as compared to Democrats who read the neutral control article (N=311, *p*<0.01, χ2 test), suggesting that the common enemy article had the opposite effect on Democrats’ identification with the national superordinate category as compared to Republicans. These results are consistent with our proposed mechanism, as quoted in the main text from Rutchik & Eccleston (2010) (*11*), who argue that “when there is a perception that subgroups do not have a shared conception of the superordinate group, appeals to the common ingroup identity made by outgroup members are likely to backfire” (pg. 111). In particular, we find evidence that Republicans and Democrats have different (and in some respects, opposing) conceptions of the superordinate category of “American” and its relation to the common enemy threat in our experiment; moreover, our results suggest that the activation of this superordinate category – and particularly among Republicans – did not lead to cross-party inclusion and identification, as popular theory suggests. Instead, our findings are consistent with the view that in highly polarized contexts, the activation of a superordinate identity may not lead to cross-party identification, and may actually exacerbate cross-party tensions (*11,12*).

This mechanism is further supported by the finding - presented in fig. S8 - that Republicans identified significantly more strongly as Americans after the assassination of Suleimani, an event that likely increased the salience of Iran as a common enemy (N=428, *p*=0.05, χ2 test). There was no significant difference in Democrats’ strength of identification with Americans before and during the Iran crisis (N=465, *p*=0.12, χ2 test). These findings are consistent with popular news accounts during the Iran crisis, which documented many prominent Republican leaders who accused Democrats of unduly lamenting the death of a dangerous Iranian general, insinuating an alliance or sympathy between Democrats and this common enemy. It is thus plausible that this Iranian threat could have increased the salience of Republicans’ sense of American identity, which excluded Democrats and framed them as being just as threatening as Iran, and indeed as potentially complicit in this common enemy threat. This is also consistent with our behavioral outcomes in fig. 4 from the main text, which show that after the Iran crisis, Republicans became even less willing to incorporate information from Democrats in a cooperative estimation task.


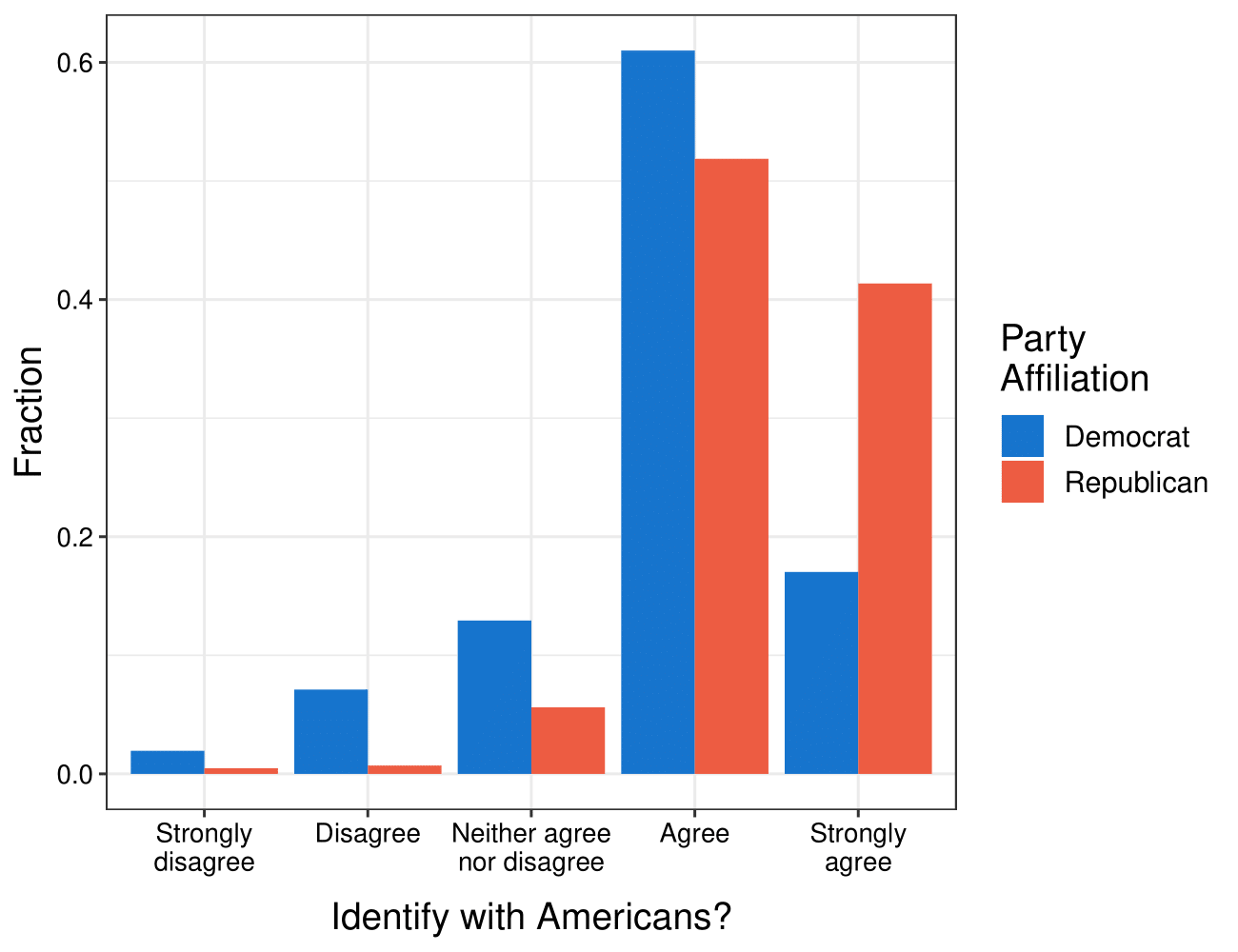


Fig. S5. Exit survey results showing the strength of identification with being American for both Democrats and Republicans; results are collapsed across conditions.


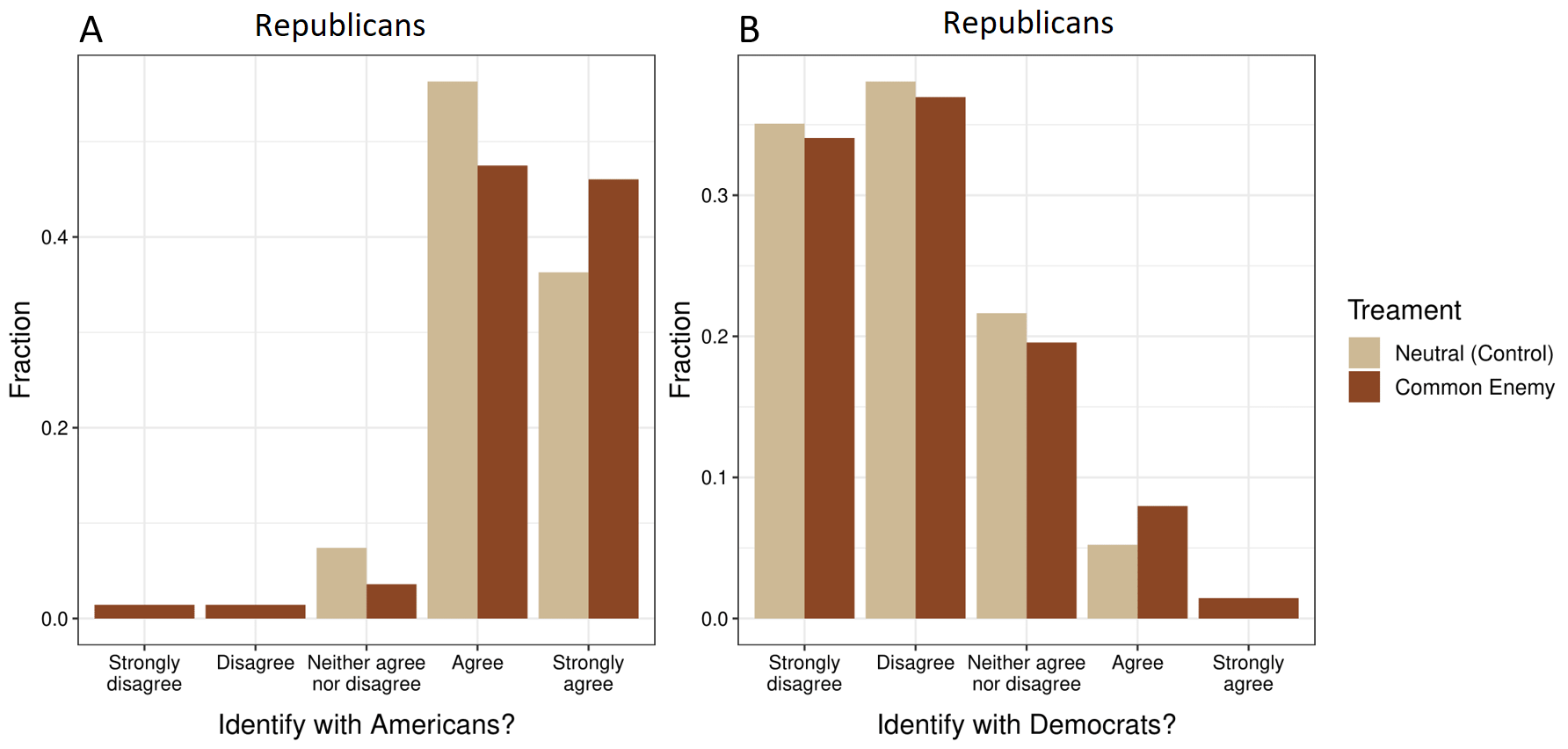


Fig. S6. Exit survey results showing Republicans’ strength of identification with (A) Americans and (B) Democrats, within both the neutral (control) condition and the common enemy condition.


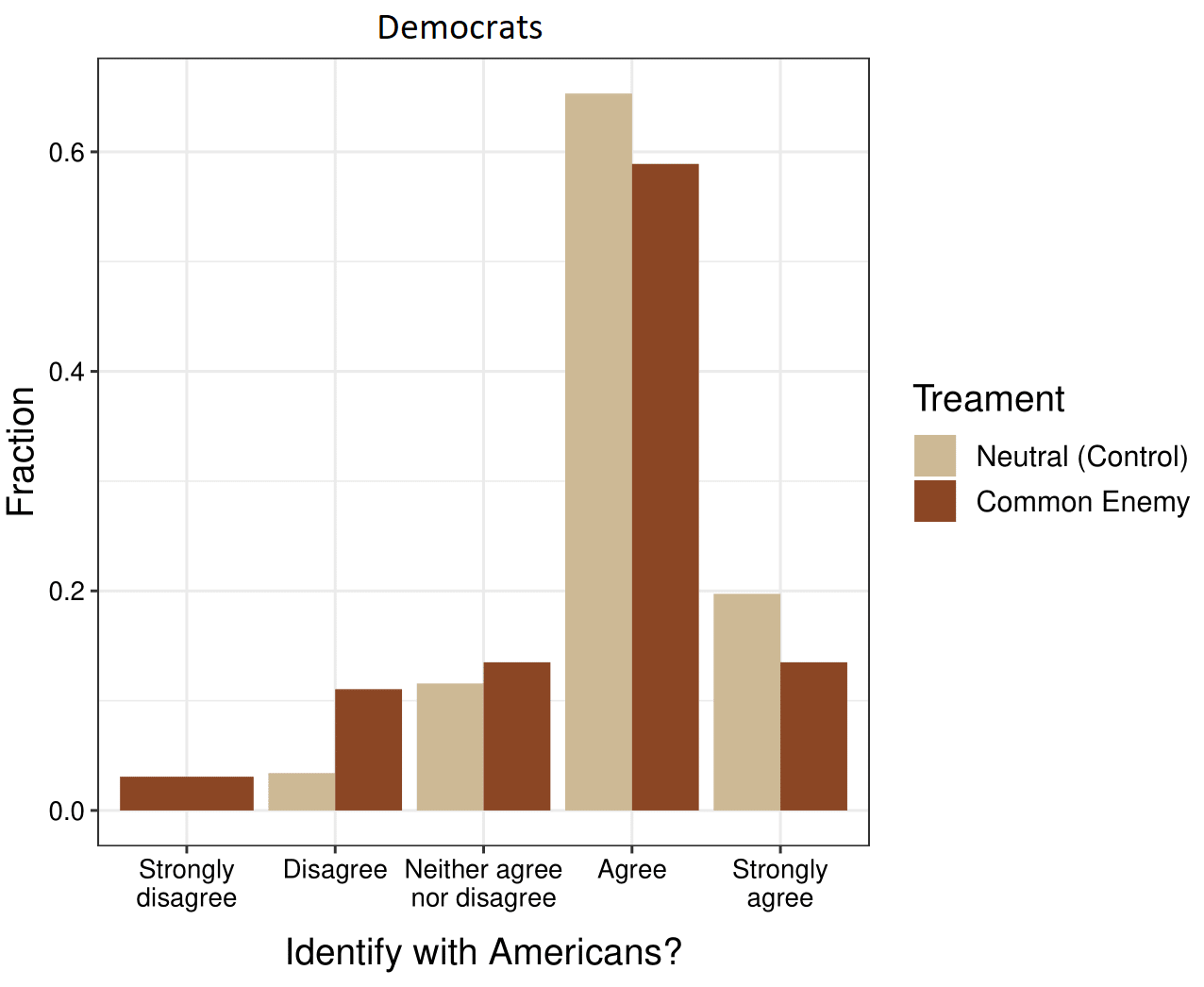


Fig. S7. Exit survey results showing Democrats’ strength of identification with Americans within both the neutral (control) condition and the common enemy condition.

**
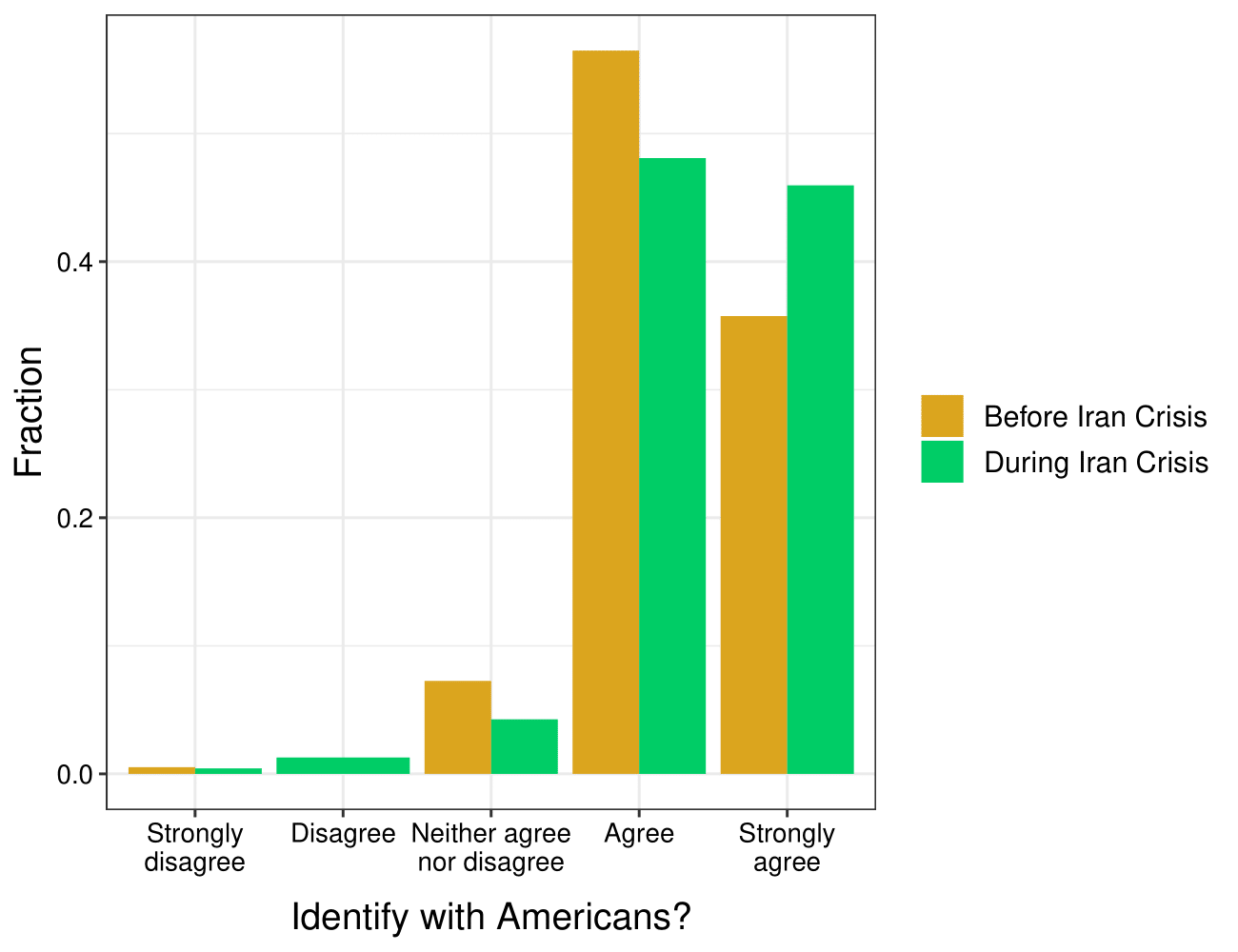
**

Fig. S8. Exit survey results showing Republicans’ strength of identification with Americans before and during the Iran crisis. Results are collapsed across priming conditions.

**SUPPLEMENTARY DISCUSSION**

**Deviations from Preregistration.** The full text of our pre-registration is available via the Open Science Framework here: <https://osf.io/eupbv/?view_only=686c0ad6d24d4cf498156c4710ad47c0>. Though we focused upon our pre-registered hypotheses about asymmetric polarization in the main text of our article, some of our predictions about this process were not supported by our analysis. For example, we initially hypothesized we would observe asymmetric polarization across both of our treatment conditions (patriotic and common enemy priming), but we only found evidence of this process in the latter condition. We also pre-registered rival hypotheses that were not supported by our findings. Based on the common ingroup identity model, we highlighted the possibility that participants in our common enemy and patriotic priming conditions would incorporate more information from the outgroup bot; this hypothesis was not supported (N=1015, p > 0.05). We made a separate set of predictions about how people would behave in response to different types of behavior by our bot. More specifically, we expected participants to cooperate more with the friendly bot (that updates its estimates towards respondents) than the stubborn bot (which does not). We did not find consistent evidence of this behavior. Since it takes more than one round for the player to discern the bot’s behavior (as either friendly or stubborn), we compare the difference in updates between the first and final estimating rounds via models that include the same controls as those reported in figure 2 from the main text of our article. These models indicate Republican participants are more likely to collaborate with the stubborn bot than the friendly bot (p < 0.01, N = 471), but we observed no significant differences in the updating behavior of democrats based on the type of bot they encountered on our online platform (p > 0.05, N = 521). In contrast, our pre-registration statement predicted two effects of partisanship. First, we tested competing hypotheses about whether Democrats or Republicans were more accommodating of the stubborn bot. However, there was no difference between Republicans and Democrats in updating towards the stubborn bot (total update from the first to final round; t(974)=3.17, p > 0.05).

Our analyses of the data also deviated from our pre-registration statement in several significant ways. In this document, we stated that we would evaluate our hypotheses by examining updating behavior within each round of estimating and between the first and last round. Yet we did not realize that a) information about the party affiliation of the bot impersonating a member of the opposing party would be redundant after the first round of updating; and b) people’s experience of the bot’s initial guess would be more consequential than subsequent rounds because of the considerable heterogeneity in respondents’ initial estimates. For these reasons, we elected to focus upon the updating behavior of respondents between the first two rounds and reported the other models in our supplementary materials (above). In our pre-registration document, we also stated that we would control for the type of bot (friendly or stubborn) in our analyses. Because we did not observe significant interactions between bot behavior and our priming conditions— and because we adopted the aforementioned binned measure of respondent’s initial estimate, which interacts with bot behavior— we elected not to include this measure in our models. We also pre-registered a mediation analysis. More specifically, we included measures of personal affect (PANAS), and ingroup and outgroup identification as potential mediators of any effect to the patriotic and common enemy primes. When conducting mediation analyses, these mediators did not predict cooperation with the outgroup bot. In retrospect, we concluded that these measures may not have been effective because they were administered after the prime and estimating task had been completed, rather than immediately after the prime. Finally, we did not pre-register our analyses of the pre and post-Iran crisis effects of the common enemy prime because it was not possible to anticipate such unprecedented events at the time we wrote our pre-registration statement.

**Additional Study Limitations.** Despite its many important contributions, our study also has several important limitations. First, we only analyzed the effect of common enemy priming in one country, and during a single historical period. It is possible that common enemies may reduce political polarization in other less polarized periods, or in countries that are not dominated by two parties. Second, our measure of social learning was limited to an anonymous, online setting where respondents could not experience shared emotions about the common enemies they were confronted with. It is possible that the realization of shared fear is a key mechanism of depolarization among members of rival political groups. Finally— though we attempted to identify a common enemy prime that was least likely to prime partisan positions— it is possible that other shared threats, such as large-scale public health pandemics, might create different effects. Despite these limitations, we hope that our study will provide a helpful basis for future research on political polarization, social identity, and the fledgling field of computational social science which routinely employs the types of online experiments used in this study to advance our understanding of large-scale human behavior.

**SUPPLEMENTARY REFERENCES**

1. Litman, Leib, Jonathan Robinson, and Tzvi Abberbock. “TurkPrime.Com: A Versatile Crowdsourcing Data Acquisition Platform for the Behavioral Sciences.” *Behavior Research Methods* 49, no. 2 (April 1, 2017): 433–42.<https://doi.org/10.3758/s13428-016-0727-z>.
2. Hauser, David J., and Norbert Schwarz. “Attentive Turkers: MTurk Participants Perform Better on Online Attention Checks than Do Subject Pool Participants.” *Behavior Research Methods* 48, no. 1 (March 1, 2016): 400–407.<https://doi.org/10.3758/s13428-015-0578-z>.
3. Watson, David, Lee Anna Clark, and Auke Tellegen. “Development and Validation of Brief Measures of Positive and Negative Affect: The PANAS Scales.” *Journal of Personality and Social Psychology* 54 (1988): 1063–70.<https://doi.org/10.1037/0022-3514.54.6.1063>.
4. Budak, Ceren, Sharad Goel, and Justin M. Rao. “Fair and Balanced? Quantifying Media Bias through Crowdsourced Content Analysis.” *Public Opinion Quarterly* 80, no. S1 (January 1, 2016): 250–71.<https://doi.org/10.1093/poq/nfw007>.
5. Levendusky, Matthew S. “Americans, Not Partisans: Can Priming American National Identity Reduce Affective Polarization?” *The Journal of Politics* 80, no. 1 (January 1, 2018): 59–70.<https://doi.org/10.1086/693987>.
6. Box-Steffensmeier, Janet M., Suzanna De Boef, and Tse-Min Lin. "The dynamics of the partisan gender gap." *American Political Science Review* 98.3 (2004): 515-528.
7. Oberski, Daniel. 2014. "survey: An R package for complex survey analysis of structural equation models." *Journal of statistical software* 57(1): 1-27.
8. Pew Research Center. “Political Polarization in the American Public,” 2014.<https://www.pewresearch.org/politics/2014/06/12/political-polarization-in-the-american-public/>
9. Jost, John T., Jack Glaser, Arie W. Kruglanski, and Frank J. Sulloway. “Political Conservatism as Motivated Social Cognition.” *Psychological Bulletin* 129, no. 3 (2003): 339–75.<https://doi.org/10.1037/0033-2909.129.3.339>.
10. Jost, John T. “The End of the End of Ideology.” *The American Psychologist* 61, no. 7 (October 2006): 651–70.<https://doi.org/10.1037/0003-066X.61.7.651>.
11. Rutchick, Abraham M., and Collette P. Eccleston. “Ironic Effects of Invoking Common Ingroup Identity.” *Basic and Applied Social Psychology* 32, no. 2 (May 19, 2010): 109–17.<https://doi.org/10.1080/01973531003738353>.
12. Klar, Samara. “When Common Identities Decrease Trust: An Experimental Study of Partisan Women.” *American Journal of Political Science* 62, no. 3 (2018): 610–22.<https://doi.org/10.1111/ajps.12366>.
